# Identification of Genes Crucial for Biological Processes in Breast Cancer Liver Metastasis Relapse

**DOI:** 10.3390/ijms25105439

**Published:** 2024-05-16

**Authors:** Tyler Kwok, Suneetha Yeguvapalli, Kumaraswamy Naidu Chitrala

**Affiliations:** Department of Engineering Technology, University of Houston, Sugar Land, TX 77479, USA; tkwok@cougarnet.uh.edu (T.K.); syeguvap@central.uh.edu (S.Y.)

**Keywords:** breast cancer recurrence, breast cancer liver metastasis, bioinformatic analysis

## Abstract

Breast cancer, when advancing to a metastatic stage, involves the liver, impacting over 50% of cases and significantly diminishing survival rates. Presently, a lack of tailored therapeutic protocols for breast cancer liver metastasis (BCLM) underscores the need for a deeper understanding of molecular patterns governing this complication. Therefore, by analyzing differentially expressed genes (DEGs) between primary breast tumors and BCLM lesions, we aimed to shed light on the diversities of this process. This research investigated breast cancer liver metastasis relapse by employing a comprehensive approach that integrated data filtering, gene ontology and KEGG pathway analysis, overall survival analysis, identification of the alteration in the DEGs, visualization of the protein–protein interaction network, Signor 2.0, identification of positively correlated genes, immune cell infiltration analysis, genetic alternation analysis, copy number variant analysis, gene-to-mRNA interaction, transcription factor analysis, molecular docking, and identification of potential treatment targets. This study’s integrative approach unveiled metabolic reprogramming, suggesting altered *PCK1* and *LPL* expression as key in breast cancer metastasis recurrence.

## 1. Introduction

Metastasis due to cancer is one of the leading causes of cancer deaths among patients with breast cancer [1]. It is known to be highly complex, involving multiple cellular mechanisms such as tumor invasion, evasion of immune surveillance, and regulation of the tissue microenvironment [2]. Among the metastases, liver metastasis is a common and devastating complication for patients with breast cancer, occurring in over 50% of those with advanced disease. Though early-stage breast cancer has a 5-year survival rate approaching 100%, this prognosis drastically declines by 26% upon diagnosis of distant organ involvement [3]. Unfortunately, there are currently no standardized therapeutic protocols explicitly tailored for BCLM due to an incomplete understanding of the molecular patterns and mechanisms governing this metastatic process. Elucidating the genetic factors that drive BCLM development and relapse following treatment is imperative to determine patient prognosis, identify the highest risk, and develop targeted therapies.

A critical first step is to define the landscape of differentially expressed genes (DEGs) between primary breast tumors and matched BCLM lesions. However, uncertainty remains regarding the key molecular biomarkers and functional pathways involved in BCLM pathogenesis. A thorough investigation of DEGs between liver metastases and the original cancer can thus provide clinically valuable information on the prognostic and therapeutic potential of these genetic alterations. In summary, comprehensive profiling of differential gene expression patterns between primary and metastatic liver tumors can facilitate a prognosis prediction, inform clinical decision-making, and reveal novel targets and strategies for treating this common yet poorly understood complication of advanced breast cancer.

## 2. Results

### 2.1. Identification of Datasets and Analysis of Differentially Expressed Genes

**Figure 1:**
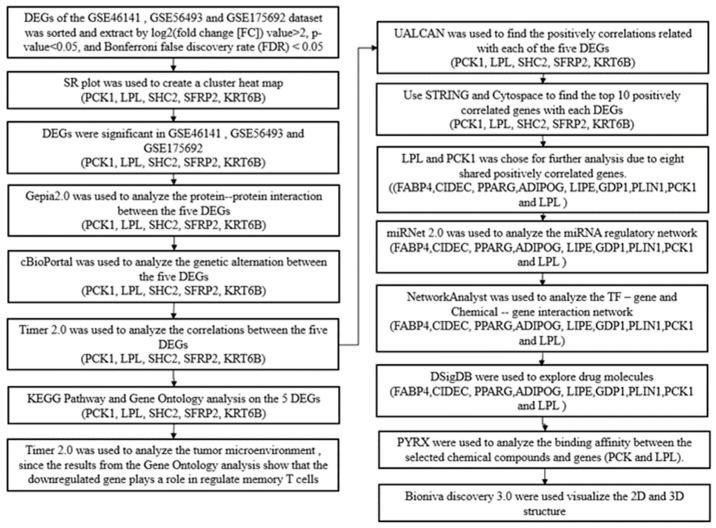
Flow chart of the bioinformatics approaches used in this study.

**Figure 2:**
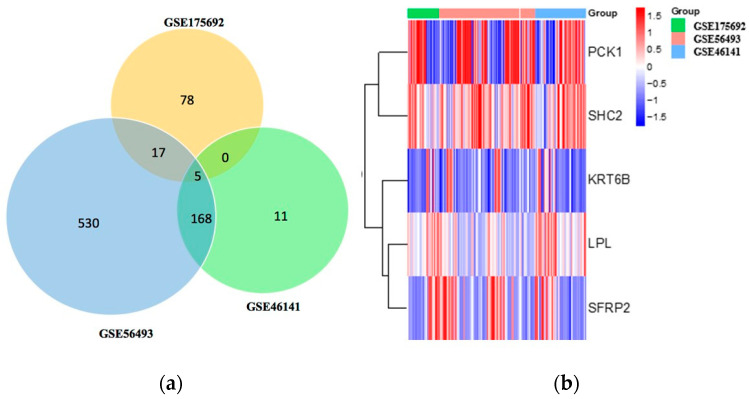
Analysis of differentially expressed genes. (**a**) Venn analysis of DEGs in the three datasets (GSE175692, GSE 56493, and GSE 46141). (**b**) Heatmap for the genes that are differentially expressed and common between the datasets GSE175692, GSE 56493, and GSE 46141.

The overall summary of the data analysis conducted in this study is shown in Figure 1. Gene expression profiles from the Gene Expression Omnibus (GEO) datasets GSE46141, GSE56493, and GSE175692 from breast cancer patients (n = 125) were selected for our study. Among these samples, pre-treatment ones (n = 83) were selected for our study. The results from the differential analysis between breast cancer local metastasis (n = 16) versus breast cancer liver metastasis (n = 11) in the GSE46141 dataset showed that 184 were significantly expressed with 154 genes being upregulated and 30 genes being downregulated. DEG analysis of the breast cancer metastasis samples from the breast (n = 19) versus the liver (n = 27) in the GSE56493 dataset showed that there were 720 genes significantly expressed with 434 being upregulated and 286 being downregulated. Further, DEG analysis of relapsed metastatic breast cancer in the breast (n = 16) versus liver (n = 27) showed that there were 100 genes significantly expressed with 59 genes being upregulated and 41 genes being downregulated. The results from the Venn analysis demonstrated that there were five genes (*PCK1*, *SHC2*, *LPL*, *SFRP2*, *KRT6B*) that overlapped between the three datasets (Figure 2a). Among the five genes, two genes (*PCK1*, *SHC2*) were upregulated and three (*LPL*, *SFRP2*, *KRT6B*) were downregulated. The expression levels of these DEGs are provided in the heatmap below (Figure 2b).

### 2.2. Identification of Genetic Alterations in the DEGs

**Figure 3:**
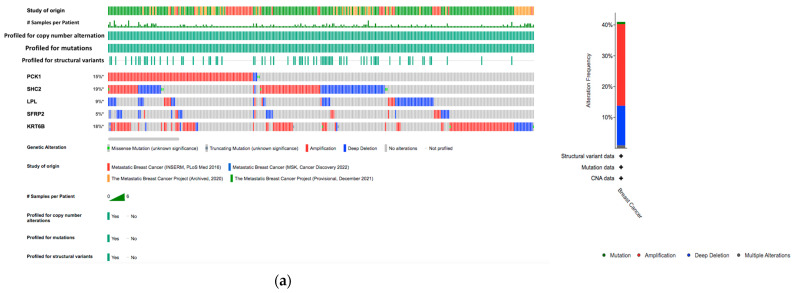

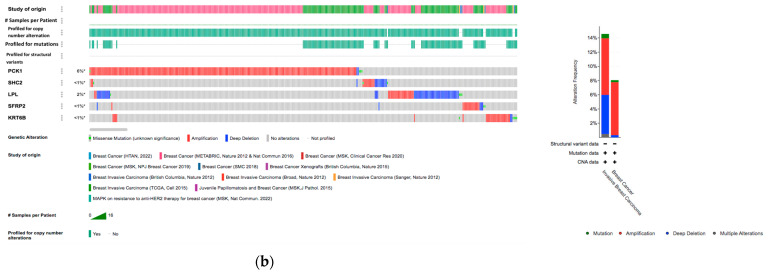
Analysis of genetic alterations related to the five DEGs across the genomic data sets (**a**) from breast cancer metastasis dataset (2197 samples/1813 patients) and an overall summary of alternations of the five DEGs in genomic data set of breast cancer metastasis; and (**b**) from breast cancer invasive data (4184 samples/4046 patients) and an overall summary of alternations of the fived DEGs in genomic data set of invasive breast cancer. * represent not all samples are profiled.

For the five genes (*LPL*, *PCK1*, *KRT6B*, *SFRP2*, *SHC2*) that were differentially expressed between the datasets, we analyzed the alterations in metastatic breast cancer and invasive breast cancer genetic profiles (Figure 3). The results from the metastatic breast samples showed that the *SHC2* gene had the highest alterations in the genetic biomarker at 19%, *KRT6B* had alterations at 18%, and *PCK1* had alterations at 15%. LPL (9%) and *SFRP2* (5%) both had less genetic alteration compared to the other DEGS. In all the genes, many alterations were mostly composed of amplification and deep deletion (Figure 3a). Conversely, there were no significant results shown in the invasive breast cancer dataset. *PCK1* had alterations at 6% and all the other genes had genetic alterations lower than 2% (Figure 3b).

### 2.3. Prognostic Information of Hub Gene Expression

**Figure 4:**
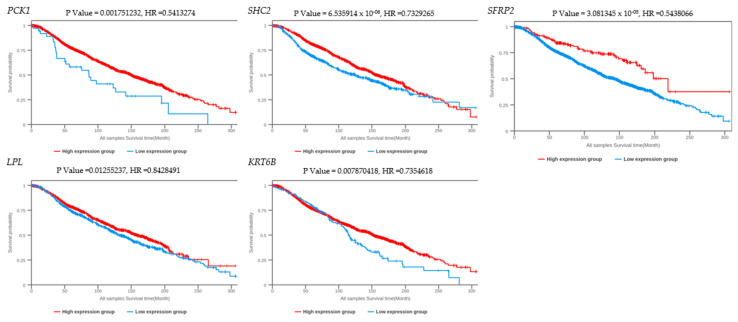
Overall survival (OS) time between five DEGs’ higher-expression level and lower-expression-level tumors in *BRCA*. The red line shows the cases with highly expressed genes and the blue line indicates the cases with lower expressed genes. HR: hazard ratio.

The overall survival (OS) and copy number variants of the DEGs were analyzed using the breast cancer integrative platform. The Kaplan–Meier plotter (Figure 4) was used to identify the survival of the five DEGs. The results showed that the expression level of *SFRP2* (HR = 0.544, P = 3.08 × 10^−05^), *SHC2* (HR = 6.54 × 10^−06^, P = 0.733), and *PCK1* (HR = 0.541, P = 0.00175) was positively correlated with the OS of patients. The expression level of *LPL* (HR = 0.843, P = 0.0126) and *KRT6B* (HR = 0.736, P = 0.00787) did not show a significant correlation to the OS of patients.

Additionally, we analyzed the expression of the DEGs for copy number variants (CNVs), gain (amplifications), and loss (deletions). The results showed that copy number aberrations were associated with four of our DEGs. The expression of *PCK1* was high when CNVs were gained; conversely, there was a high expression of *LPL*, *SHC2*, and *SFRP2* when CNVs were lost. There was no significant change in expression in CNVs for *KRT6B*. (Supplementary Figure S1). Furthermore, we performed a survival analysis on the copy number variant associated with the OS of the gene expression, but there was no significant impact for all of the genes (Supplementary Figure S2). The presence or absence of these CNVs associated with the gene expression changes was found to be non-significant with the overall survival.

**Figure 5:**
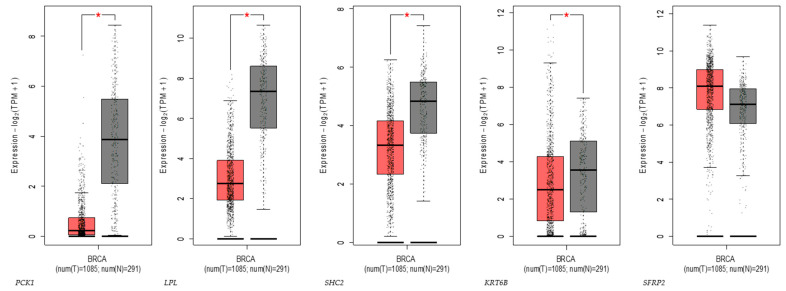
Expression of genes in breast invasive carcinoma; red bars represent expression in cancer tumors and the grey bars represent expression in normal patients. * Represents statistical significance at a *p*-value < 0.05.

The GEPIA2 database was used to verify the expression levels of the DEGs in tumor and normal tissues. As shown in Figure 5, *SHC2*, *LPL*, *PCK1*, and *KRT6B* were all under-expressed, and only *SFRP2* was highly expressed in the breast tumor. Additionally, the expression levels of the DEGs in different stages of BC are shown in Supplementary Figure S3. As listed in Supplementary Table S1, *PCK1*, *LPL*, and *SFRP2* play a role in breast cancer’s initial and developmental stages, and *KRT6B* is exclusively related to the developmental stages.

### 2.4. Gene Network Analysis

**Figure 6:**
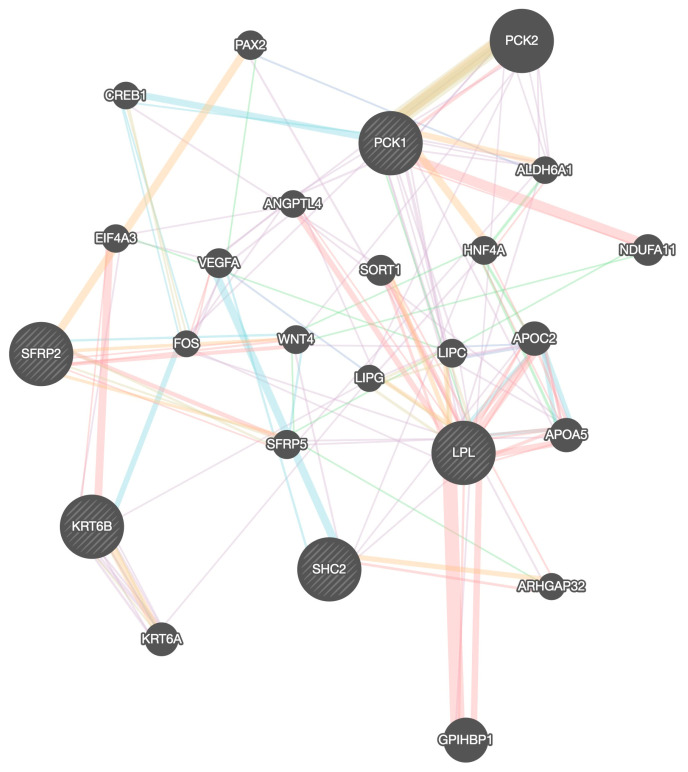
Protein–protein interaction analysis of DEGs. Red line represents physical interaction, purple line represents co-expression, orange line represents predictions, blue line represents co-localization, green line represents genetic interaction, and light blue line represents pathways.

To analyze the protein–protein interaction of the DEGs, we used Gene mania (Figure 6). *LPL*, *PCK1*, and *SHC2* were co-expressed, and there was a genetic interaction between *LPL* and *PCK1*. We used Signor 2.0 to visualize the signaling pathways between the co-expressed genes with a confidence score greater than 0.5 (Supplementary Figure S4). We validated the gene relationship between *LPL* and *PCK1* with Timer 2.0 (Supplementary Figure S5A). Both genes had a significant positive correlation in all breast cancer subtypes. The result also showed that downregulated genes *SFRP2* and *LPL* were positively correlated in all breast cancer subtype groups (Supplementary Figure S5B). The analysis conducted through Gepia 2.0 further demonstrated the expression of *SFRP2* and *LPL* in different breast cancer subtype groups (Supplementary Figure S6). The results showed that *LPL* is still under-expressed in tumor cells and that *SFRP2* is overexpressed in all HER2, Luminal A, and Luminal B subtype groups, and under-expressed in the Basal_like subtype group.

### 2.5. Identification of Positive Correlated Genes

To gain insights into the potential interacting partners and biological pathways associated with the DEGs, a series of integrated bioinformatics analyses were performed. First, the UALCAN online tool [4] was used to identify genes exhibiting a positive Pearson correlation (coefficient > 0.4) with the expression levels of the five DEGs across the breast cancer TCGA datasets. Next, the resultant gene list was uploaded to the STRING database [5] to construct a protein–protein interaction (PPI) network (Supplementary Figure S7) incorporating the DEGs and their correlated genes. Cytoscape 3.10.2 software with the CytoHubba application was then used to analyze the topology of the STRING-generated PPI network and we selected the top 10 hub genes for *SHC2 (PHPT1*, *MRPL41*, *ATP5F1D*, *RHOT2*, *NME3*, *TUBGCP6*, *SNRNP70*, *WDR90*, *THEM259*, *E4F1)*, *PCK1 (PPARG*, *ACACB*, *PLIN1*, *LIPE*, *ADIPOQ*, *GPD1*, *CIDEC*, *PCK1*, *LPL*, *FABP4)*, *LPL (PPARG*, *ADIPOQ*, *CAV1*, *LIPE*, *LPL*, *PLIN1*, *CIDEC*, *FABP4*, *CD36*, *GDP1)*, *KRT6B (TRIM29*, *KRT14*, *KRT15*, *PKP1*, *KRT6B*, *DSG3*, *KRT16*, *KRT17*, *SERPINB5*, *DSC3)*, and *SFRP2 (COL1A1*, *MMP2*, *DCN*, *LUM*, *TGFB1*, *BGN*, *COL1A2*, *POSTN*, *FN1*, *COL3A1)* ranked by their degree of interaction (Supplementary Figure S8A). Gene ontology (GO) term (Supplementary Figure S8C) and KEGG pathway (Supplementary Figure S8B) enrichment analyses of these 10 hub genes were conducted using the DAVID database. This revealed that two selected genes, phosphoenolpyruvate carboxykinase 1 (*PCK1*) and lipoprotein lipase (*LPL*), were commonly enriched in pathways involved in fatty acid and energy metabolism regulation, including PPAR signaling, AMPK signaling, regulation of lipolysis in adipocytes, and thermogenesis. Integrating interactome and pathway databases in this manner provided novel biological insights into the molecular mechanisms potentially implicated in breast-to-liver metastasis by DEGs and their correlated network hubs (*FABP4*, *CIDEC*, *PPARG*, *ADIPOG*, *LIPE*, *GDP1*, *PLIN1*, *PCK1*, *LPL*).

### 2.6. Analysis of Gene to miRNA and Transcription Factor Interaction

A network was constructed to analyze the relationship between the target miRNA and transcription factors with the network hub genes (*FABP4*, *CIDEC*, *PPARG*, *ADIPOG*, *LIPE*, *GDP1*, *PLIN1*, *PCK1* and *LPL*). miRNet 2.0 was applied to screen the targeted miRNAs of the hub genes. As illustrated in Supplementary Figure S9, the interaction network consisted of 70 nodes and 88 edges. The interactive hub genes that most miRNAs targeted were *LPL* (degree score = 30) and *PPARG* (degree score = 29), and the highest interactive miRNAs was hsa-mir-27a-3p (degree score = 5). From the results, we also identified four miRNAs targeting both the *PCK1* and *LPL*. The miRNA hsa-mir-27a-3p targeted *LPL*, *PCK1*, *CIDEC*, *PLIN1*, and *PPARG*; miRNA has-mir-124-3p targeted *LPL*, *PCK1*, and *CIDEC*; and both miRNAs hsa-mir-10b-5p and hsa-mir-200b-3p targeted *LPL* and *PCK1*. Transcription factor (TF) enrichment analysis was performed to identify over-represented TF binding sites in promoters of the hub genes using a network analyst (Supplementary Figure S10). The results showed that the transcription-regulated network of hub genes included 30 nodes and 33 edges. *NFKB1* and *RELA* were the highest interactive transcription factor, with a degree of 3. Among the transcription factors, *SP1* was the only regulator that was common in both *LPL* and *PCK1*, and it mainly plays a role in transcriptional mis-regulation in cancer.

### 2.7. Identification of Potential Treatment Targets

**Figure 7:**
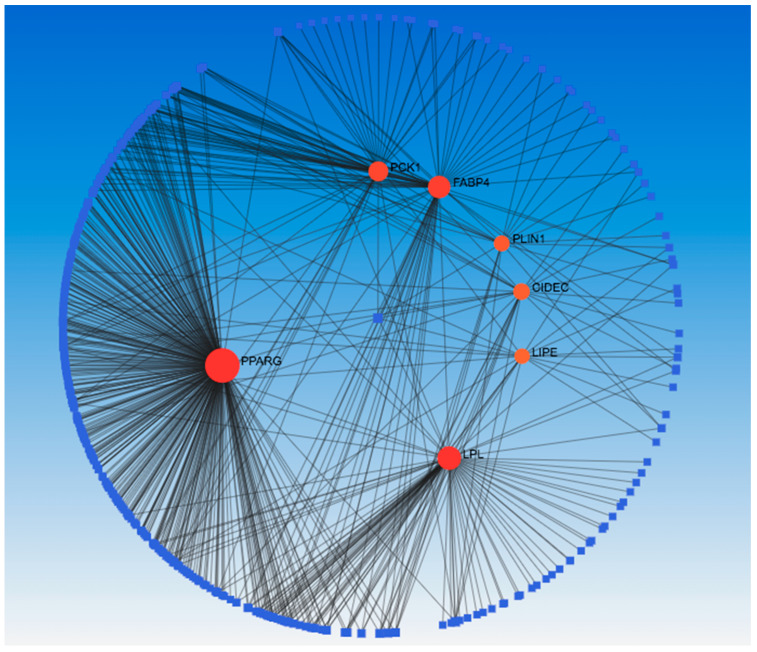
Identification of interaction between hub genes and chemicals. Network showing the hub genes, which are shown in red color nodes, whereas chemicals are shown in blue color nodes. Lines refer to the interaction between the hub genes and chemicals.

A network analysis was performed to identify the chemical to hub genes interaction (Figure 7). The results showed that there were 447 nodes and 634 edges with bis(4-hydroxyphenyl)sulfone, Dexamethasone, and rosiglitazone having the highest degree (7) of interaction within the hub genes. To drug targets for the hub genes, we submitted them to the DSigDB database (Table 1) and ranked them based on their adjusted *p*-value. Our results showed that Triflumizole and Rosiglitazone CTD were the most significant drugs of the hub gene in the DSigDB database, followed by IBMX BOSS, formic acid BOSS, IBMX CTD 00007018, Bisphenol A diglycidyl ether CTD 00000976, oleic acid BOSS, glycerol BOSS, D-glucose BOSS, and insulin BOSS.

### 2.8. Molecular Docking for Protein–Chemical Interaction

The four common chemicals (bis(4-hydroxyphenyl)sulfone, Dexamethasone, Triflumizole, and Rosiglitazone) that showed the highest interaction between the hub genes were used to analyze their respective interactions with the genes *LPL* and *PCK1*. Our results (Supplementary Table S2) showed that Rosiglitazone had the highest binding affinity (−8.4) with 1NHX, with three hydrogen bonds at THR A:339, ASN A:292, and VAL A:335; three pi-alkyl interactions seen at LYS A:290,VAL A:335, and ARG A:87; and one pi-cation and one carbon hydrogen bond formed at ARG A:405 and ASP A:311 (Supplementary Figure S11b). Dexamethasone (−8.1) showed the second highest binding affinity towards 1NHX. Dexamethasone formed two hydrogen bonds at ARG A:405 and one hydrogen bond at LYS A:290 and CYS A:288. There was also a Halogen (Fluorine) at ARG A:405 (Supplementary Figure S11c). Bis(4-hydroxyphenyl)sulfone(−7.5) formed five hydrogen bonds at HIS A:264, ASP A:311, SER A:286, AIA A:287, and CYS A:288; and one pi-Alkyl and Pi sigma interaction was formed at LYS A:290 and VAL A:335, respectively (Supplementary Figure S11d). Triflumizole (−7.0) formed two hydrogen bonds with PHE A:530 and ASN A:533. Pi-pi T-shaped formed a bonding with PHE A:525 and pi-pi stacked formed a bonding with PHE A:530, while one fluorine interaction and carbon–hydrogen bond was formed with CYS A:288 and TRP A:516, respectively. Four pi-Alkyl interactions were formed at TRP A:516, PHE A:517, PHE A:525, and PHE A:530 (Supplementary Figure S11a). Rosiglitazone(−7.7) also had the highest binding affinity when it interacted with 6e7k, following Triflumizole (−7.5), Dexamethasone (−7.3), and bis(4-hydroxyphenyl)sulfone (−7.3). As seen in Supplementary Figure S12b, Rosiglitazone had three pi-alkyl interactions at ILE A:221, LYS A:265, and VAL A:84; and pi-pi stacked, pi-pi t-shaped, pi-cation and carbon hydrogen bonds were formed at TYR A:121,TRP A:82, LYS A:265, and HIS A:268, respectively. Triflumizole (Supplementary Figure S12a) had two halogens (Flourine) at ILE A:221 and HIS A:268, and two hydrogen bonds at SER A:159 and HIS A:268. Six pi-alkyl were formed with PHE A:212, HIS A:268,LYS A:265, and ILE A:221; and two were formed at TYR121. Five alkyls were seen at VAL A:264 and PRO A:187, and two were seen at ILE A:221. Three carbon hydrogen bonds were formed with HIS A:268, SER A:159, and PRO A:187. Dexamethasone and bis(4-hydroxyphenyl)sulfone both had the same binding affinity. Dexamethasone formed two hydrogen bonds at THR A:379 and GLU A:298. There was one Halogen (Florine) and unfavorable acceptor–acceptor interaction at GLU A:298 and SER A:390, respectively (Supplementary Figure S12c). Bis(4-hydroxyphenyl)sulfone formed two hydrogen bonds with HIS A:268 and TYR A:158, and two Pi-Pi t-shaped were formed with TRP A:82. One Pi-cation and Pi- Sulfur was formed at LYS A:265 and HIS A:268. Two Pi-Alkyl interactions were formed at LYS A:265 and ILE A:221 (Supplementary Figure S12d). All the chemical compounds were tested for their druglikeness. The results showed that these compounds had druglikeness (Supplementary Figure S13).

### 2.9. Gene Ontology (GO) and Functional Enrichment Analysis

**Figure 8:**
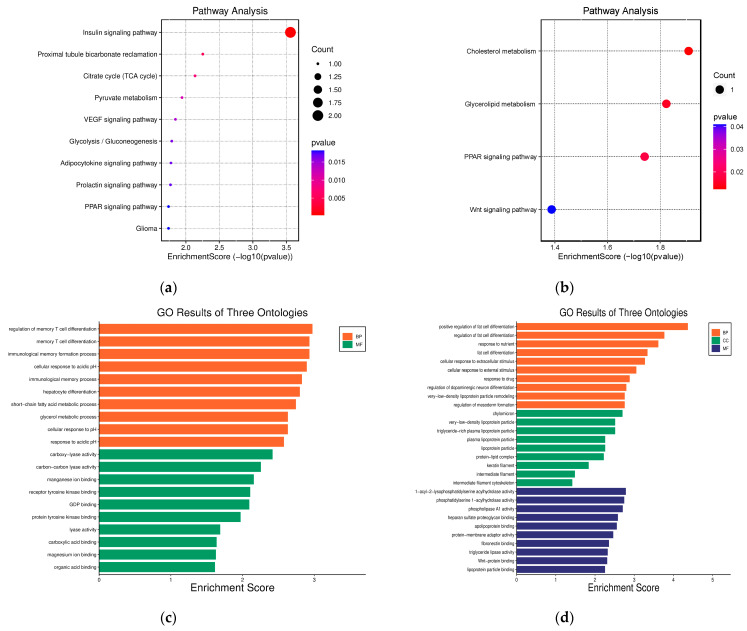
Function enrichment analysis for DEGs. KEGG pathway analysis with (**a**) upregulated and (**b**) downregulated DEGs. GO analysis with (**c**) upregulated (**d**) and downregulated DEGs.

The Database for Annotation, Visualization, and Integrated Discovery (DAVID; version 6.8) was utilized to perform the functional enrichment analysis of the five most prevalent DEGs. The Kyoto Encyclopedia of Genes and Genomes (KEGG) pathway analysis identified the molecular pathways enriched among the up- and downregulated genes. The two upregulated DEGs were significantly enriched in the insulin signaling pathway (Figure 8a). Meanwhile, the three downregulated DEGs were expressed in several metabolic pathways (Figure 8b), including Cholesterol metabolism, Glycerolipid metabolism, and Peroxisome proliferator-activated receptor (PPAR) signaling pathway. Gene ontology (GO) term enrichment was also conducted across biological processes, cellular components, and molecular functions. The upregulated DEGs (Figure 8c) were primarily associated with regulating the memory T-cell differentiation for biological processes. Considering that the upregulated genes (*SHC2* and *PCK1*) have a role in the regulation of memory T-cell differentiation and the immunological memory formation process, we examined the correlations between the upregulated genes and infiltrated immune cells. Based on Supplementary Figure S14 we can see that both *SHC2* and *PCK1* were positively correlated with the infiltrating levels of NK cells, activated mast cells, and macrophages M2. In contrast, downregulated DEGs (Figure 8d) were mainly involved in the positive regulation of adipocyte differentiation from precursor cells, response to nutrients, and cellular response to extracellular stimuli. These results from DAVID highlight the dysregulation of insulin signaling and metabolic pathways that may contribute to the pathobiology of breast cancer liver metastasis according to the expression patterns of the consistent DEGs between datasets.

## 3. Discussion

Recent studies and research indicate that breast cancer is currently the most frequent type of cancer among females, exceeding “lung cancer as the leading cause of cancer incidence” in the U.S. and globally [3]. Roughly 20% of individuals diagnosed with breast cancer will encounter a recurrence and 50–70% of metastatic breast cancer cases involve the liver [6]. Moreover, studies have shown that there is a poor prognosis for breast cancer metastasis to the liver, with the median survival rate being only 2–3 years [7]. Although BCLM is relatively common, there is a scarcity of specialized therapeutic options available for patients. This signifies that there is a dire need for the development of interventions in this area, which could only be made possible by rigorous research and an in-depth understanding of molecular processes underlying breast cancer to allow progress in the early diagnosis and treatment of the condition [8]. Therefore, our bioinformatic research aimed to identify potential biomarkers and assist in predicting the accurate behavior of the illness, and aid in the creation of targeted therapeutic approaches. Researching biomarkers can provide insights into the molecular processes that underlie the spread of cancer, its metastasis, and its resistance to treatment. This information can further our understanding of cancer biology and aid in the creation of novel targeted treatments.

In this study, we used proven online bioinformatics tools to investigate possible biomarkers for the diagnosis of breast cancer and therapeutic drugs. Three datasets were selected from the GEO database due to their relevance to our research topic; GSE56493 and GSE46141 were run on the Rosetta/Merck Human RSTA Custom Affymetrix 2.0 microarray platform. GSE175692 was retrieved which was run on the nCounter Breast Cancer 360 Panel platform. We identified five DEGs common to all three GEO datasets, which included two upregulated genes and three downregulated genes. The upregulated genes were mainly involved in the insulin signaling pathway (Figure 8a), which is proven to be an important factor for breast cancer prognosis [9]. The downregulated genes did not have significant results in pathways enrichment (Figure 8b), but the gene ontology results showed that they play a role in the regulation of fat cell differentiation (Figure 8d). Genetic alteration analysis was performed on the DEGs in metastatic breast samples (Figure 3). We observed major genetic alterations in *PCK1*, *SHC2*, and *KRT6B*, emphasizing the role of these genes in tumor behavior modification as well as the response to treatment. These changes, such as amplification and deep deletion, remain significant for understanding the complex mechanisms of breast cancer evolution. In addition, the CNVs analysis (Supplementary Figure S1) showed the copy number aberrations associating with gene expression. With the assistance of the BCIP platform, we found that the expression of *PCK1* is higher when CNVs are gained. This may imply that an increase in expression changes the cancer cell metabolism. When tumors are coming from different organs, the combination of genetic alteration and environmental stress would determine the expression level of *PCK1*. For instance, a high expression of gene *PCK1* in CNV gain influences the inducement of retrograde carbon flow from gluconeogenesis [10]. As a result, there would be a decrease in glutathione levels, while ROS production would be enhanced, thereby supporting hypoxic breast cancer growth. Since *PCK1* performs an anti-oncogenic role in gluconeogenic organisms, its gains in CNVs would thus affect breast cancer metastasis in the liver due to its tumor-promoting role. Specifically, *PCK1* enhances liver metastatic growth by facilitating pyrimidine nucleotide biosynthesis in hypoxia, a significant feature in the liver microenvironment. Further studies may be focused on exploring how *SHC2* CNV loss can impact the prognosis of breast cancer metastasis.

Previous research has found that *SHC2* is coding for the SHC-coding transforming protein 2, which associates with the cancer’s start and later progression [11]. *KRT6B* regulates EMT and cytoskeletal dynamics, which were found to support cancer cell migration, proliferation, invasion, and metastasis [12]. *SFRP2*, a protein in the SFRP family, was found to act as a counter-direction signal from the Wnt/β type pathway, and is associated with various cancer incidences, including breast cancer [13]. However, further research is needed to analyze the mechanism of the genes *SHC2*, *KRT6B*, and *SFRP2* in breast cancer liver metastasis. To gain a further insight into the functions and potential role that the five DEGs have in breast cancer local metastasis and in metastasis in liver, we collected genes that were positively related with them (with a score >0.4). We created a PPI among all the positively correlated genes (Supplementary Figure S8a) for each DEG and identified the hub genes that can be used for gene ontology and KEGG pathway enrichment analysis (Supplementary Figure S8b,c). The KEGG pathway results showed that the top-degree genes that were positively correlated with SHC2 were mainly associated with cholesterol metabolism, glycerol lipid metabolism, the PPAR signaling pathway, and the WNT signaling pathway. The top-degree genes that were positively correlated with KRT6B were mainly associated with Staphylococcus aureus infection and the estrogen signaling pathway, and the top-degree genes that were positively correlated with SFRP2 were mainly associated with Proteoglycans in cancer, the AGE-RAGE signaling pathway in diabetic complications, Amoebiasis, the Relaxin signaling pathway, and diabetic cardiomyopathy. Among the pathways listed, previous studies have shown that the PPAR signaling pathway, WNT signaling, and Proteoglycans in cancer are directly correlated with breast cancer metastasis. Dysregulation of PPARγ signaling is linked to tumor development in breast [14] and Proteoglycans can influence cell growth by interacting with growth factors via their core proteins or their GAG chains. The genetic mutation-driven activation of Wnt signaling is the key factor in breast cancer metastasis [15]. Wnt is a regulator of EMT and it induces EMT in increasing tumor progression. A high expression level of Wnt occurs during the progression of tumor cells and promotes metastasis [16].

When we analyzed each of the DEGs and their positively correlated hub genes, we also found that the pathway analysis and gene ontology results were closely related between *PCK1* and *LPL* (Supplementary Figure S8b,c). Therefore, we took the top-degree shared hub genes between *PCK1* and *LPL* to explore and create a network analysis. It is controversial whether *PCK1* plays an oncogenic or tumor suppressor function in various human cancers. *PCK1* has antitumorigenic effects in gluconeogenic organ cancers (liver and kidney) but tumor-promoting effects in non-gluconeogenic organ malignancies. Prior studies have shed light on *PCK1*’s hijacking function and mechanisms in cancer of the colon, hepatocellular carcinoma, breast, kidney, etc. Higher *PCK1* activity allows metastatic breast cancer cells to engage in gluconeogenesis, thereby generating glucose and glyceroneogenesis [17]. This allows for the creation of biosynthetic operations. This metabolic adaptation enables the cells to survive in a liver microenvironment. *PCK1* expression allows breast cancer cells that have metastasized to the liver to create a disadvantage, driving key metabolic fluxes. *LPL* plays a major role in influencing breast cancer liver metastasis because it affects the rates of lipid metabolism. Breast cancer is influenced by metabolic abnormalities that affect its onset and progression, especially increased lipid synthesis and uptake. However, depending on the patient’s hormone status and the stage of the disease, lipid metabolism has different effects on breast cancer. The maintenance of the proliferation and survival of breast cancer cells depends on the dysregulation of fatty acid metabolism [18]. Liver cancer cells are more likely to take up and use fatty acids when *LPL* expression is higher. As a result, there is an increased potential for breast cancer cells to proliferate and spread into liver tumors.

In our miRNA and hub gene analysis, we identified four miRNAs of interest (Supplementary Figure S9), which included hsa-miR-27a-3p, hsa-miR-124-3p, hsa-miR-10b-5p, and hsa-miR-200b-3p. These targeted miRNAs are important regulators involved in the development and metastasis of breast cancer that are mainly associated with the change in expression of *PCK1* and *LPL*. Additionally, has-miR-27a-3p is a two-sided regulator of the transformation of metabolism and metastasis [19]. Evidence has shown that it can reduce the expression of *PCK1*, which is an essential enzyme in gluconeogenesis, and shift the metabolism toward favoring cell proliferation and metastasis. The silencing of the *PCK1* gene by hsa-miR-27a-3p may trigger a metabolic reprogramming of cancer cells which would make them take over and become more resistant and invasive when under nutrient starvation, which is a typical situation in the tumor microenvironment.

On the other hand, it was found that hsa-miR-124-3p functions as a tumor suppressor in various cancers such as breast cancer. It has been demonstrated that *LPL* downregulation and targeting by this drug is critical for cancer cell liposuction, which is crucial for providing the energy and molecules required for their fast growth and reproduction. Hsa-miR-124-3p can block the *LPL* function and therefore inhibit the lipid metabolism and the cell energy homeostasis of the breast cancer cells, which would be a strategy to control its metastasis [20]. Hsa-miR-10b-5p, which is famous for its pro-metastatic effects, was suggested as a *PCK1* regulation directly. Over-expression of the precursor of hsa-miR-10b-5p is directed towards the downregulation of *PCK1*, activating glycolysis over gluconeogenesis; enhancing the Warburg effect, a metabolic hallmark of cancer cells; and facilitating the metastasis of breast cancer cells.

Moreover, hsa-miR-200b-3p, a member of the miR-200 family, plays a crucial role in controlling Epithelial–Mesenchymal Transition (EMT), a key process in cancer metastasis progression [21]. While there is not a direct link between *PCK1* and *LPL* expression, they could contribute to EMT, thereby promoting metabolic changes and lipid metabolism. These alterations may enhance the metastatic capability of breast cancer cells.

A TF network of hub genes was constructed to explore the molecular networks associated with breast cancer. In Supplementary Figure S10, the results show that Sp1 was the only transcription factor that is shared between *PCK1* and *LPL*, and it is mainly involved in transcriptional misregulation in cancer. Studies have shown that Sp1 can directly bind to the promoter region of the *PCK1* [22] and controls the proliferation of breast cancer cells by interacting with insulin-like growth factors I receptor [23], enhancing their transcriptional activity. Therefore, targeting Sp1-mediated transcriptional regulation may be a potential therapeutic strategy, but more investigations are needed to understand TFs and hub gene interactions in regulating breast cancer liver metastasis.

We performed a chemical-to-gene interaction (Figure 8) of the nine hub genes that were identified in the studies. Rosiglitazone, Dexamethasone, and bis(4-hydroxyphenyl)sulfone were shown to interact closely with the hub genes. Rosiglitazone, a pharmacological compound, binds to PPARγ (peroxisome proliferator-activated receptor gamma), a nuclear receptor protein controlling gene expression [24]. The activation of PPARγ by Rosiglitazone leads to the overexpression of several genes crucial in lipid and glucose metabolism. For instance, it triggers increased activity of *LPL*, encoding enzymes to break down triglycerides from lipoproteins, thereby enhancing their breakdown in the blood. Additionally, Rosiglitazone induces the expression of *PCK1*, encoding an enzyme in the pathway producing glucose from non-carbohydrates, ultimately enhancing the hepatic glucose output. A further analysis in drug databases revealed Rosiglitazone CTD 00003139 (Table 1) as a potential treatment, validating the importance of Rosiglitazone in the treatment of recurrent breast cancer metastasis in the liver. Further research needs to be performed for Dexamethasone and bis(4-hydroxyphenyl)sulfone in breast cancer liver metastasis recurrence. The precise effect of bis(4-hydroxyphenyl)sulfone on breast cancer remains unclear, while dexamethasone was found to be a double-edged sword during breast cancer progression and metastasis [25]. At lower concentrations, Dexamethasone inhibits the growth and metastasis of breast cancer tumors, while higher concentrations may promote breast cancer progression.

To explore candidate drugs by molecular docking simulations, *PCK1* and *LPL* were utilized as the target receptors to analyze their interactions with the chemicals (bis(4-hydroxyphenyl)sulfone, Dexamethasone, Triflumizole, and Rosiglitazone). The molecular analysis results showed that bis(4-hydroxyphenyl)sulfone, Dexamethasone, Triflumizole, and Rosiglitazone have a high binding affinity with the target receptors (Supplementary Figures S11 and S12). These chemicals could be essential for discovering potential drug candidates for the treatment of recurrent breast cancer metastasis to the liver and should be subjected to further testing.

Our study has some limitations, since it relies solely on bioinformatics analysis and lacks experimental verification; more clinical trials and research are needed to validate the miRNAs, TFs, chemical compounds, and potential drugs. Despite using algorithm-based scientific methodologies, our research serves to predict the potential biomarkers and therapeutic targets for treating recurrent breast cancer metastasis to the liver.

## 4. Materials and Methods

### 4.1. Identification of Datasets and Analysis of Differentially Expressed Genes

In this study, a combined bioinformatics approach was used to find essential differentially expressed genes (DEGs) and describe the functions of these genes by combining different data sets. Gene expression datasets related to advanced local and liver metastasis breast cancer were obtained from the National Center for Biotechnology Information’s Gene Expression Omnibus (GEO) database (https://www.ncbi.nlm.nih.gov/geo/, accessed on 9 November 2023) [26]. Local and liver metastasis samples were retrieved from the GSE46141 [27] (n = 30) and GSE56493 [27,28] (n = 43) datasets. Their respective metastatic recurrence information was retrieved from the GSE175692 [29] dataset. Differential gene expression (DEG) analysis between the following groups was performed: (1) breast cancer local versus liver metastasis, (2) breast cancer metastasis in breast anatomical site versus breast cancer metastasis in liver anatomical site, (3) relapsed metastatic breast cancer in breast organ versus liver. We used the following cut-off threshold values to identify DEGs: log2(fold change [FC]) value > 2, *p*-value < 0.05, and Bonferroni false discovery rate (FDR) < 0.05. To identify the specific and commonly dysregulated genes among the DEGs identified in each of the datasets, we performed a Venn diagram analysis.

### 4.2. Clustering and Analysis of Identified DEGs

To classify the analyzed samples based on gene expression profiles and to observe the overall gene expression patterns in each condition of the datasets, we performed hierarchical clustering using the Cluster heatmap option in the SR plot (https://www.bioinformatics.com.cn/srplot, accessed on 26 November 2023), which employs *pheatmap* R package [30]. To compare the expression of identified genes in each of the breast cancer types and the normal tissue at a threshold of *p*-value < 0.05, we used GEPIA 2.0 (Zhang Lab, Peking University, Beijing, China, Version 2) (http://gepia2.cancer-pku.cn/) [31], a web-based tool that utilizes data from the TCGA and Genotype-Tissue Expression (GTEx) databases [32] (accessed on 9 December 2023). To analyze the prognostic value of DEGs, we analyzed the copy number variants (CNV) followed by survival analysis using the breast cancer integrated platform (http://www.omicsnet.org/bcancer/, accessed on 13 January 2024) [33] using the default settings.

### 4.3. Identification of Genetic Alterations in the DEGs

To identify the genetic alterations in the DEGs, we used cBioprtal (https://www.cbioportal.org/, accessed on 18 January 2024) [34]. We selected the following breast datasets in cBioPortal for analyzing the genomic alterations in the identified DEGs: Metastatic Breast Cancer (INSERM, PLoS Med 2016); Metastatic Breast Cancer (MSK, Cancer Discovery 2022); The Metastatic Breast Cancer Project (Archived, 2020); The Metastatic Breast Cancer Project (Provisional, December 2021); Breast Cancer (HTAN, 2022); Breast Cancer (METABRIC, Nature 2012 and Nat Commun 2016); Breast Cancer (MSK, Clinical Cancer Res 2020); Breast Cancer (MSK, NPJ Breast Cancer 2019); Breast Cancer (SMC 2018); Breast Cancer Xenografts (British Columbia, Nature 2015); Breast Invasive Carcinoma (British Columbia, Nature 2012); Breast Invasive Carcinoma (Broad, Nature 2012); Breast Invasive Carcinoma (Sanger, Nature 2012); Breast Invasive Carcinoma (TCGA, Cell 2015); Juvenile Papillomatosis and Breast Cancer (MSK, J Pathol. 2015); and MAPK on resistance to anti-HER2 therapy for breast cancer (MSK, Nat Commun. 2022).

### 4.4. Immune Cell Infiltration Analysis of DEGs

To explore correlations between the DEGs and identify the clinical relevance of tumor–immune infiltrations we used the TIMER 2.0 (Li Lab and Xu Lab, University of Texas, MD Anderson Cancer Center, Houston, TX, USA, Version 2) (http://cistrome.org/TIMER/, accessed on 8 January 2024) [35] database. Using TIMER 2.0, we conducted univariate and multivariate Cox regression analyses using variables such as tumor purity, tumor stage, and hub gene expression.

### 4.5. Identify Positive Correlated Genes

To determine whether a statistically significant linear relationship existed between the DEGs and other potential genes, we used UALCAN (https://ualcan.path.uab.edu/, accessed on 2 January 2024) [4]. The tool utilizes TCGA datasets to measure the Pearson correlation between genes given their expression level in breast invasive carcinoma. If a coefficient value lied between 0.4 and 1, then a medium to strong correlation exists.

### 4.6. Gene to miRNA Interaction

To identify miRNA regulatory network for our hub genes in humans, we used miRNet 2.0 (Xia Lab, McGill University, Montreal, QC, Canada, Version 2) (https://www.mirnet.ca, accessed on 3 February 2024) [36], a platform that uses 14 different databases (TarBase, miRTarBase, miRecords, miRanda- S mansoni only, miR2Disease, HMDD, PhenomiR, SM2miR, PharmacomiR, EpimiR, starBase, TransmiR, ADmiRE, and TAM 2.0), miRanda, and TarPmiR to predict genomic targets for microRNAs.

### 4.7. Network Analysis on Hub Genes

We used the NetworkAnalyst (https://www.networkanalyst.ca/, accessed on 8 February 2024) [37] online tool to visualize a hub gene–transcription factor (gene-TF) interaction network, and protein–chemical (gene-chemical) interaction. To construct a gene-TF network, the TF and gene target data were derived from TRRUST [38], a curated database of human transcriptional regulatory networks. To create gene–chemical interaction network, we used the Comparative Toxicogenomics Database [39], a database that provide information about chemical–gene/protein interactions, chemical–disease, and gene–disease relationships.

### 4.8. Construction of Gene Networks and Protein-Protein Interactions

To identify the functionally related genes to our query genes and to perform protein–protein interaction analysis, we used the GeneMANIA (https://genemania.org/, accessed on 22 December 2023) [40] prediction server, STRING online analysis tool (http://www.string-db.org/, accessed on 22 December 2023) [5], and CytoHubba plugin in Cystoscape [41]. In GeneMANIA, we selected Homo sapiens as the target organism and interaction score > 0.4 for our analysis. In CytoHubba, we selected the top 10 genes ranked by degree for our analysis. To identify the signaling relationship associated with the up- and downregulation of the genes, we used Signor 2.0 (https://signor.uniroma2.it/APIs.php, accessed on 15 December 2023) [42].

### 4.9. Identification of Potential Treatment Targets

To identify potential treatment targets, the DEGs were analyzed using the Drug Signature Database (DSigDB), sourced from the *Enrichr* platform (https://amp.pharm.mssm.edu/Enrichr/, accessed on 12 February 2024) [43]. Enrichr provided detailed visualizations of the common functions associated with the input genes, exploring drug molecules associated with the DEGs.

### 4.10. Analyzing the Unique Ligands and Their Respective Binding to DEGs

We accessed the RCSB Protein Data Bank (PDB) (https://www.rcsb.org) [44] to obtain the three-dimensional structure of *PCK1* (PDB ID: 1NHX) and *LPL* (PDB ID: 6E7K). To prepare the protein, we used Discovery Studio Visualizer 3.0 (BIOVIA, Dassault Systèmes, San Diego, CA, USA, Version 3) (https://discover.3ds.com/discovery-studio-visualizer-download, accessed on 24 March 2024) [45,46] to remove the water and heteroatoms compounds, and add polar hydrogens to the protein structure and save it in PDB format. Then, we used PubChem (https://pubchem.ncbi.nlm.nih.gov/, accessed on 24 March 2024) [46] to download the structure of the chemical compounds [Triflumizole (Pubchem ID: 91699), bis(4-hydroxyphenyl)sulfone (Pubchem ID: 6626), Dexamethasone (Pubchem ID: 5743), and rosiglitazone (Pubchem ID: 77999)], and used SwissADME (http://www.swissadme.ch/index.php, accessed on 25 March 2024) [47] to obtain the pharmacokinetics, druglikeness, and medicinal chemistry friendliness information of the chemicals. The selected chemical compounds and the newly modified protein were provided as input into the Virtual Screening software interface PyRx (https://pyrx.sourceforge.io/, accessed on 28 March 2024) [48] for docking studies. To analyze the result from the PyRx interface, we entered the relevant docking output files back to Discovery Studio Visualizer 3.0 to determine the interaction between the respective ligands and the receptors.

### 4.11. Gene Ontology (GO) and Functional Enrichment Analysis

Functional annotation and pathway enrichment analyses of the genes were performed using the Database for Annotation, Visualization, and Integrated Discovery (DAVID; v6.8, http://david.ncifcrf.gov, accessed on 26 November 2023) database [49]. Gene ontology (GO) term enrichment was performed using the enrichment bubble option in the SR plot (https://www.bioinformatics.com.cn/srplot, accessed on 10 November 2023)**,** which uses the ggplot2 [50] function in the R package.

## 5. Conclusions

In conclusion, this study utilized an integrative bioinformatics approach to uncover critical genes and pathways involved in breast cancer liver metastasis. By comparing gene expression profiles across multiple datasets, five robust and consistent differentially expressed genes (DEGs) were identified between primary tumors and liver metastases. Further pathway and network analysis on these DEGs and correlated interacting partners revealed the enrichment of metabolic processes related to lipid and glucose metabolism. In particular, the genes *PCK1* and *LPL* emerged as central hubs exhibiting altered expression.

## Figures and Tables

**Table 1:** Potential drug treatment targets with clinical application identified for hub genes.

Name	*p*-Value	Adjusted *p*-Value	Combined Score
Triflumizole CTD 00002280	3.728 × 10^−15^	3.158 × 10^−12^	118,549.00
Rosiflitazone CTD 00003139	2.628 × 10^−13^	1.110 × 10^−10^	11,263.85
IBMX BOSS	2.049 × 10^−12^	5.771 × 10^−10^	20,983.21
Formic acid BOSS	4.897 × 10^−11^	1.034 × 10^−8^	5525.67
IBMX CTD 00007018	1.826 × 10^−10^	3.086 × 10^−8^	5723.06
BISPHENOL A DIGLYCIDYL ETHER CTD 00000976	8.713 × 10^−10^	1.227 × 10^−7^	11,104.17
Oleic acid BOSS	3.661 × 10^−9^	4.419 × 10^−7^	3127.80
Glycerol BOSS	7.426 × 10^−9^	7.492 × 10^−7^	2604.39
D-glucose BOSS	8.503 × 10^−9^	7.492 × 10^−7^	2514.27
Insulin BOSS	9.207 × 10^−9^	7.492 × 10^−7^	2462.76

## Data Availability

Publicly available datasets were analyzed in this study.

## Supplementary Materials

The following supporting information can be downloaded at https://www.mdpi.com/article/10.3390/ijms25105439/s1.
