# Supplementary material for: Identification of Genes Crucial for Biological Processes in Breast Cancer Liver Metastasis Relapse
Source: Int J Mol Sci. 2024 May 16;25(10):5439. doi: 10.3390/ijms25105439 (PMC11122209; doi:10.3390/ijms25105439)
Supplement: Supplementary file 1 [file ijms-25-05439-s001.zip › FInal Supplementary Figures- revision 1.pdf]

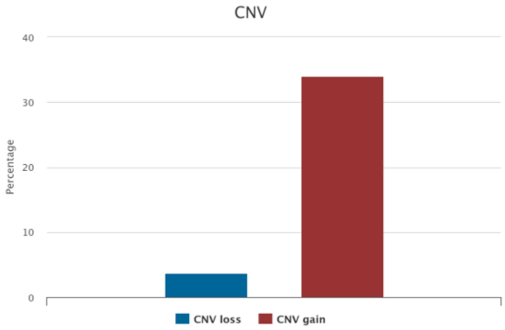

PCK1

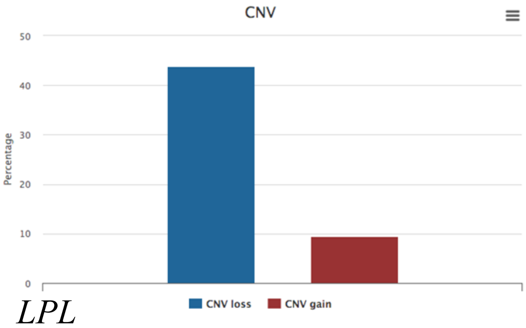

LPL

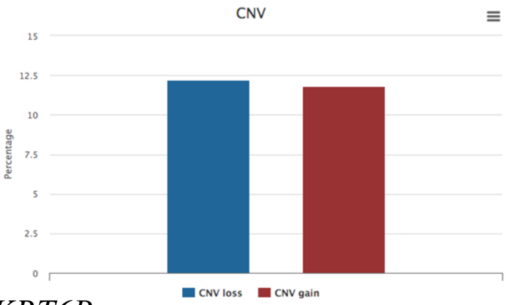

KRT6B

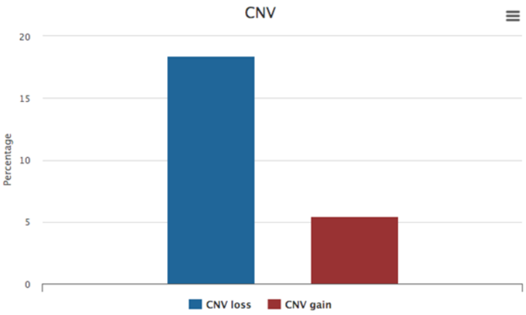

SFRP2

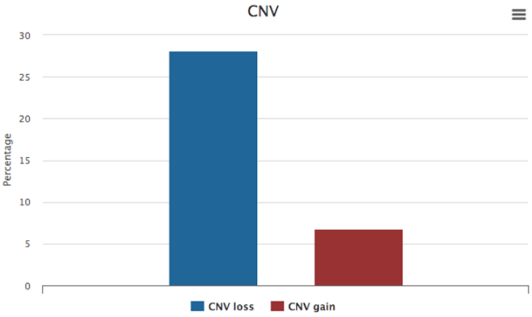

SHC2

Supplementary Figure S1. Copy number variation analysis on different expression level of the genes using the BCIP database

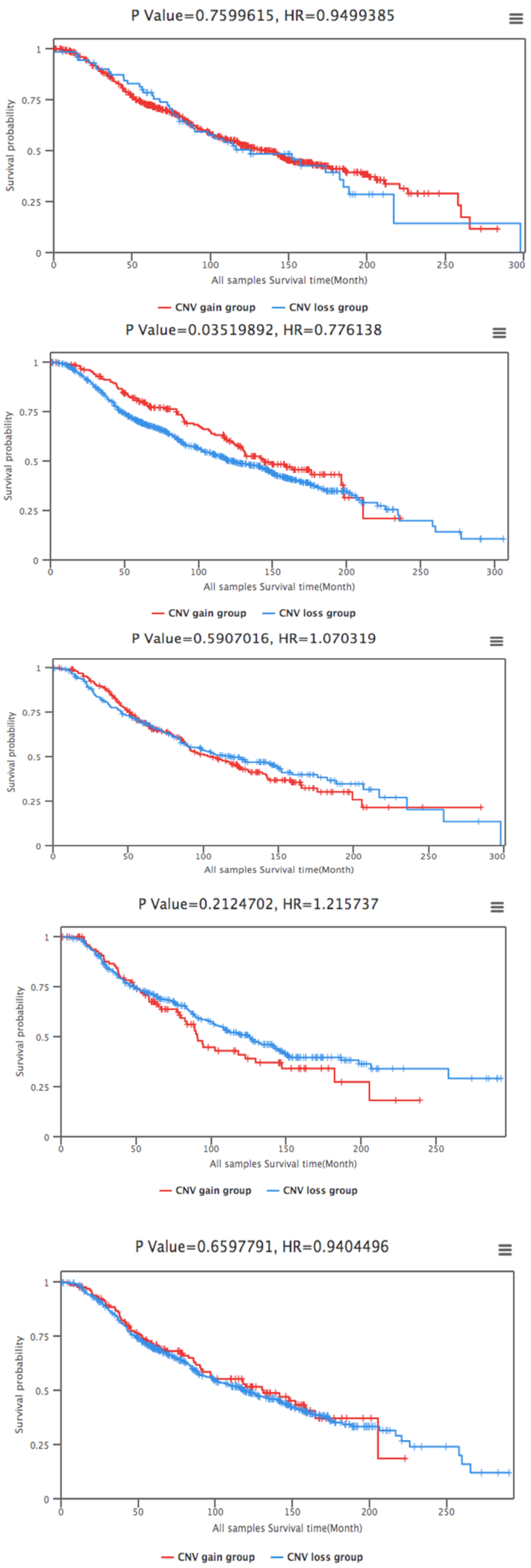

(b)

Supplementary Figure S2. Overall survival of the genes associated with CNV using the BCIP database

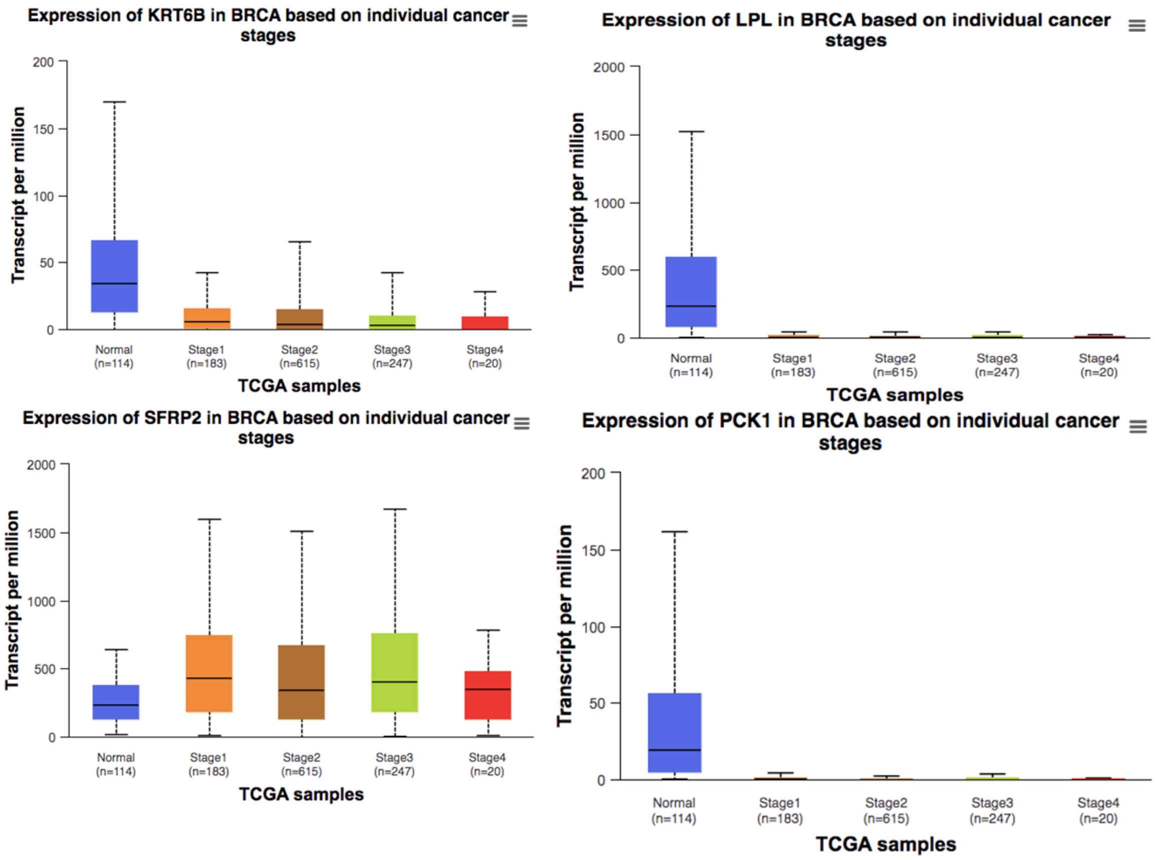

Supplementary Figure S3. Verifying the five DEGs expression based on the individual pathological breast cancer stage.

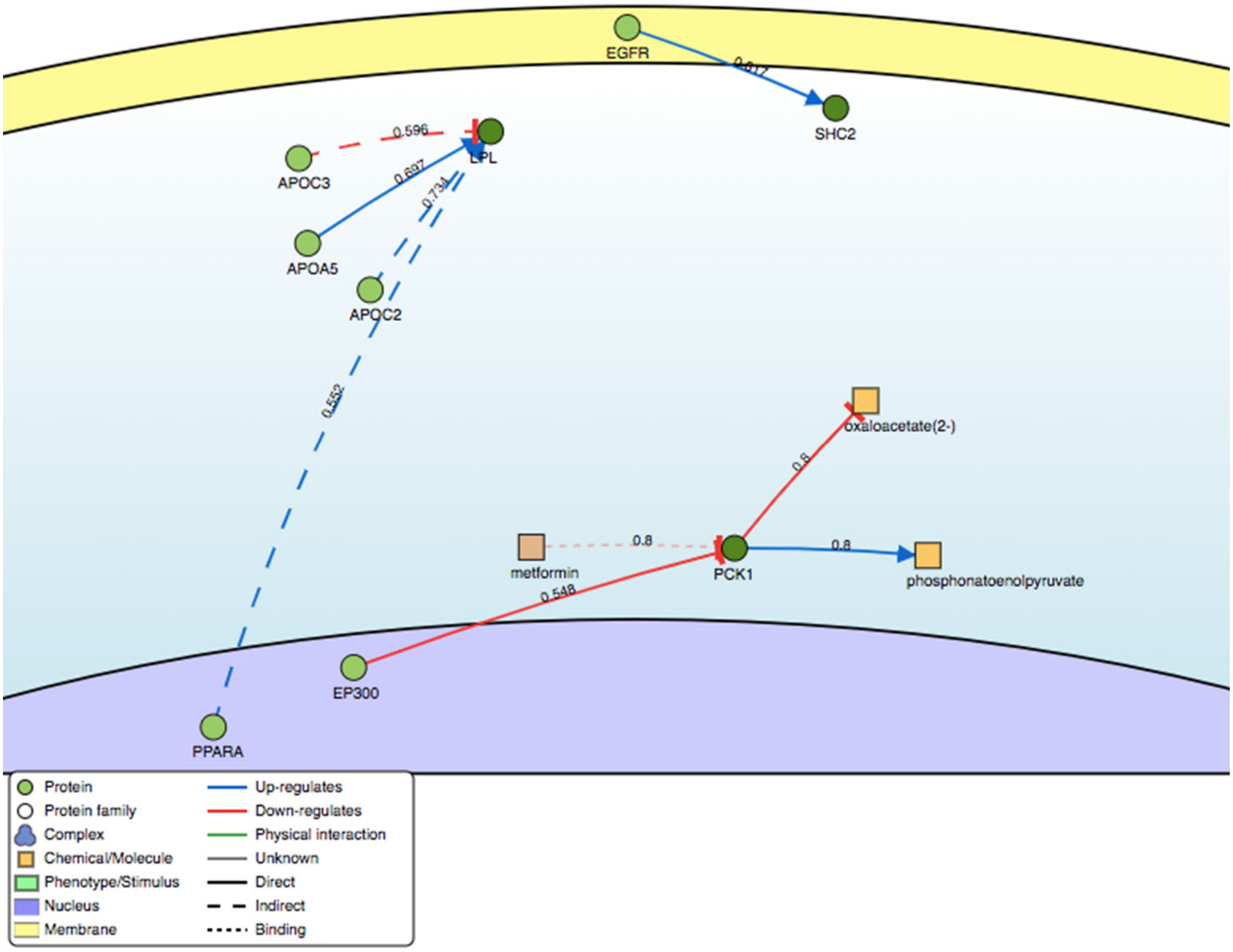

Supplementary Figure S4. Network analysis showing the shortest paths linking the genes *SHC2, PCK1,* and *LPL* with a confidence score of 0.5 and above. Activated paths are colored in blue and inhibited paths are colored in red.

A)

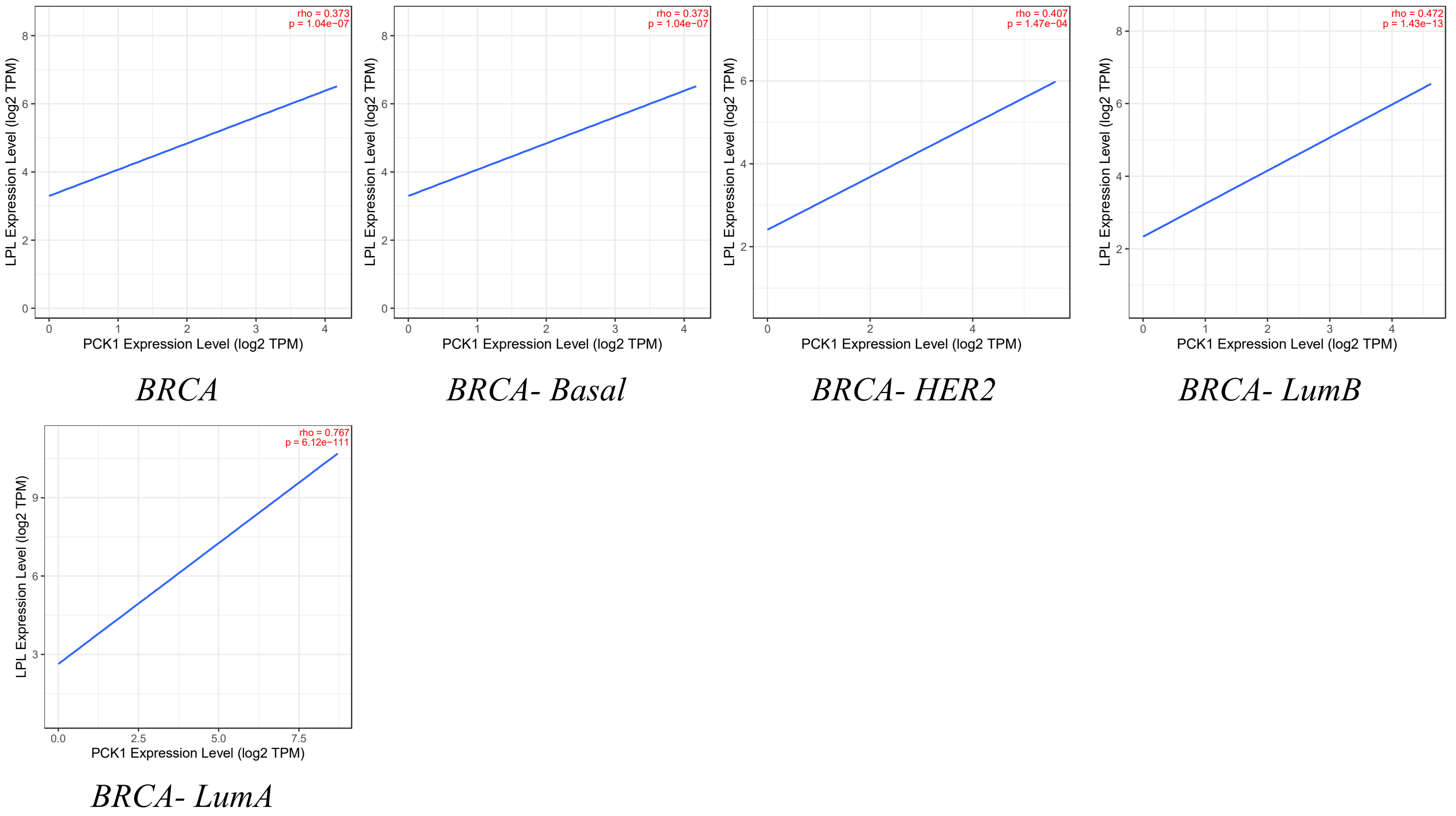

B)

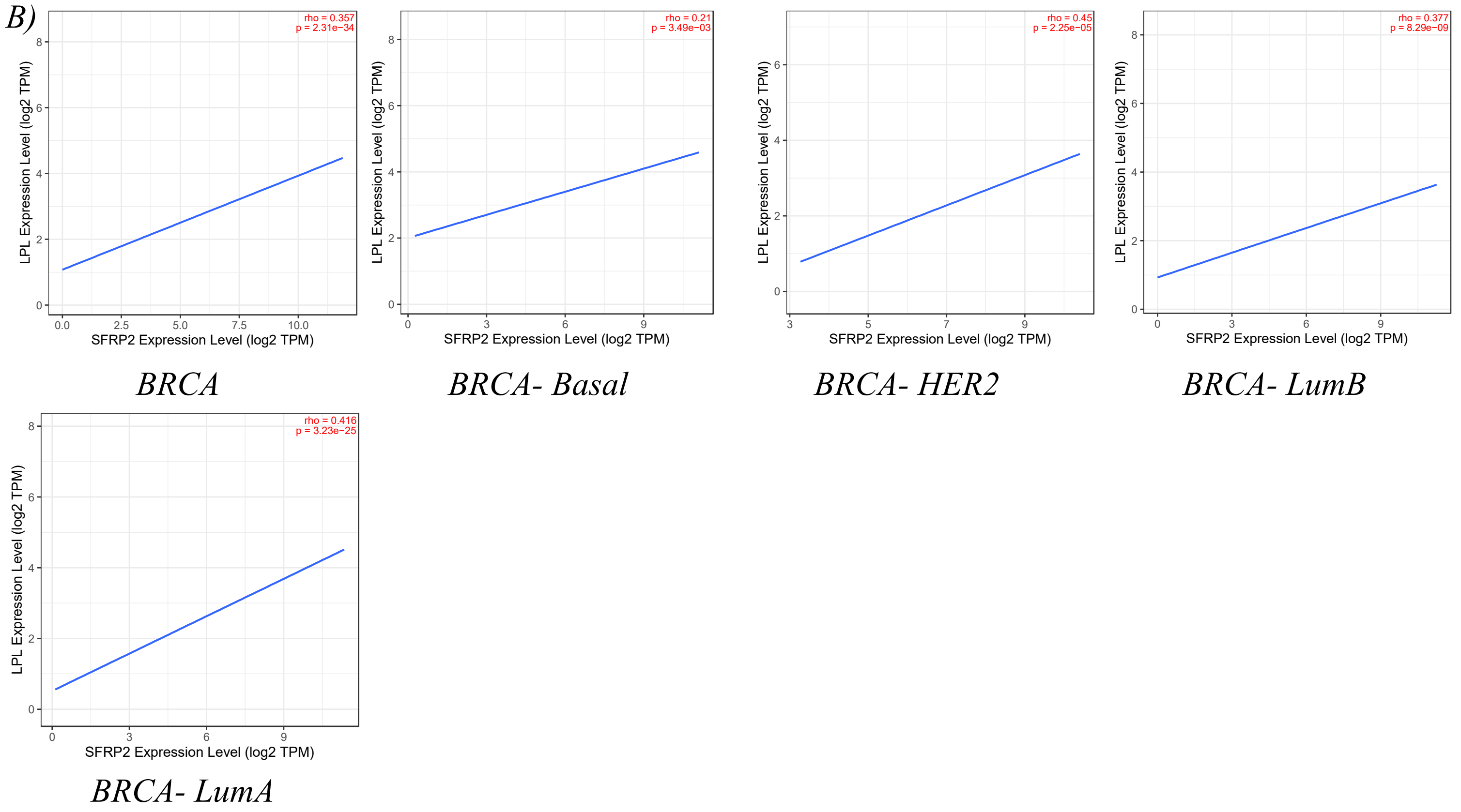

Supplementary Figure S5. Correlation analysis of the expression of two DEGs with different breast cancer types using TIMER2.0. (A) Correlation of the expression of LPL with SFRP2 level in all breast cancer subtypes. (B) Correlation of the expression of LPL with PCK1 level in all breast cancer subtypes.

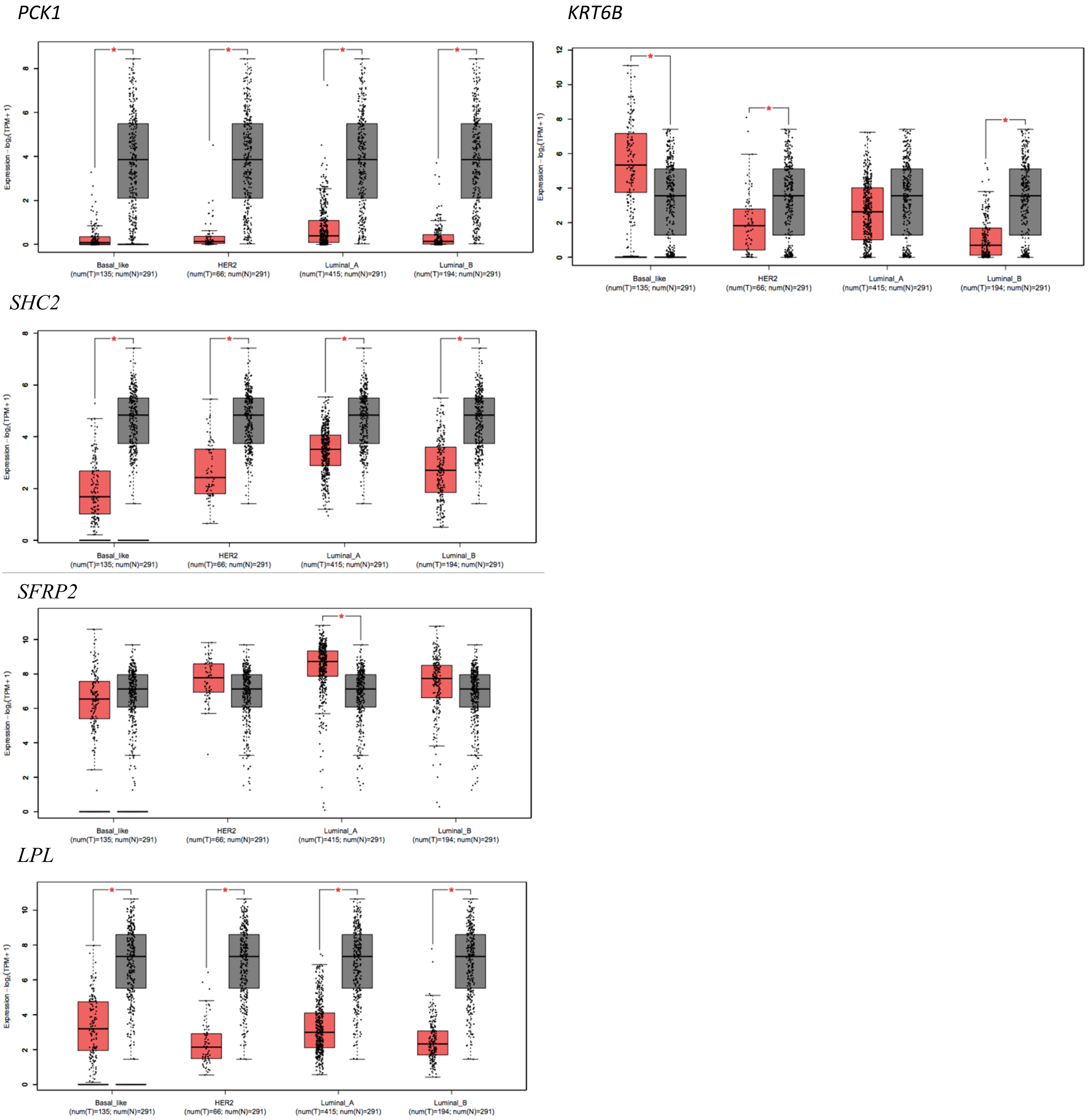

Supplementary Figure S6. Expression of Genes in different Breast cancer subtypes; Red bars represent expression in Cancer tumors and the grey bars represent expression in normal patients. *represents statistical significance at a p-value < 0.05.

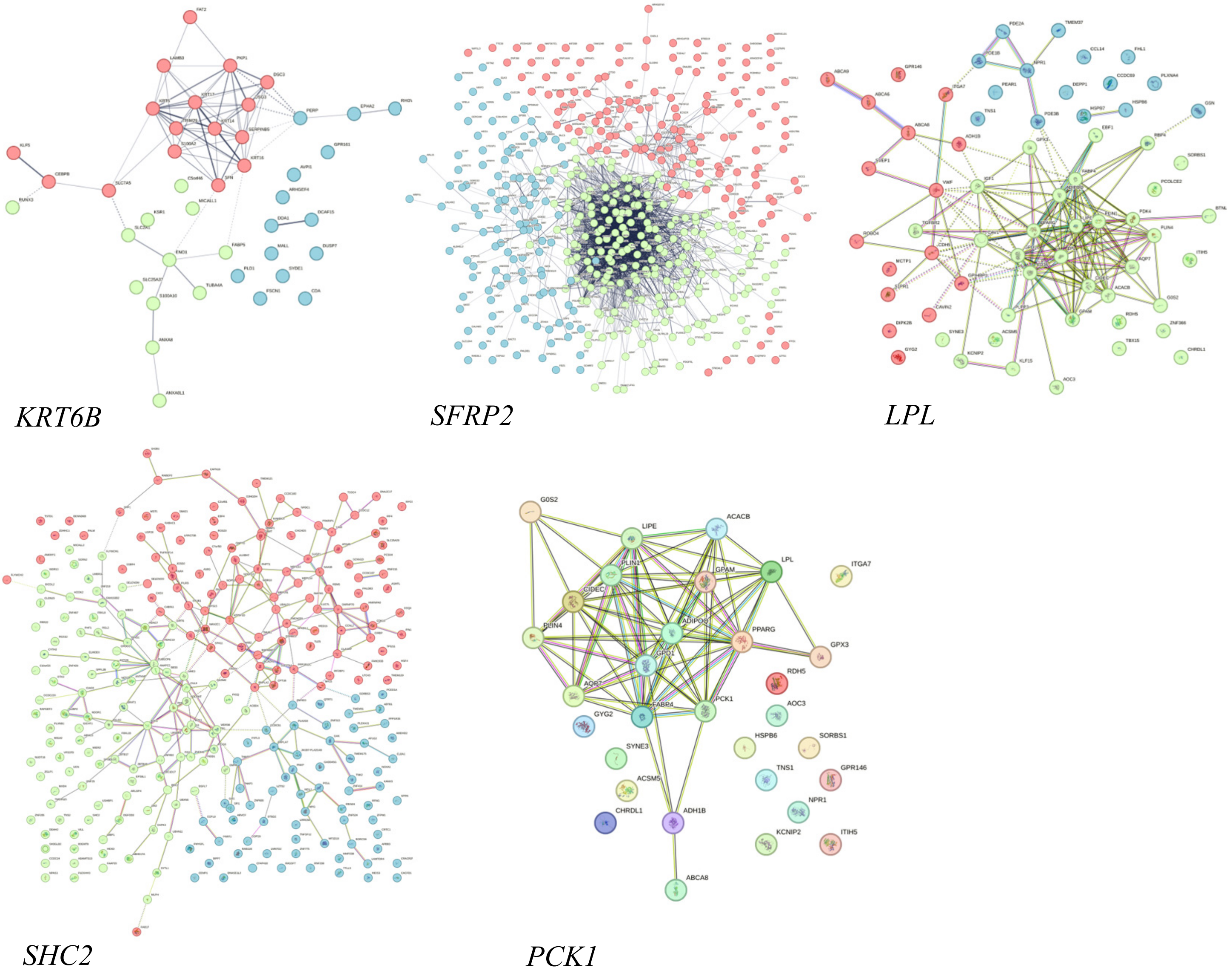

Supplementary Figure S7. PPI network of the genes that are positively correlated with the DEGs

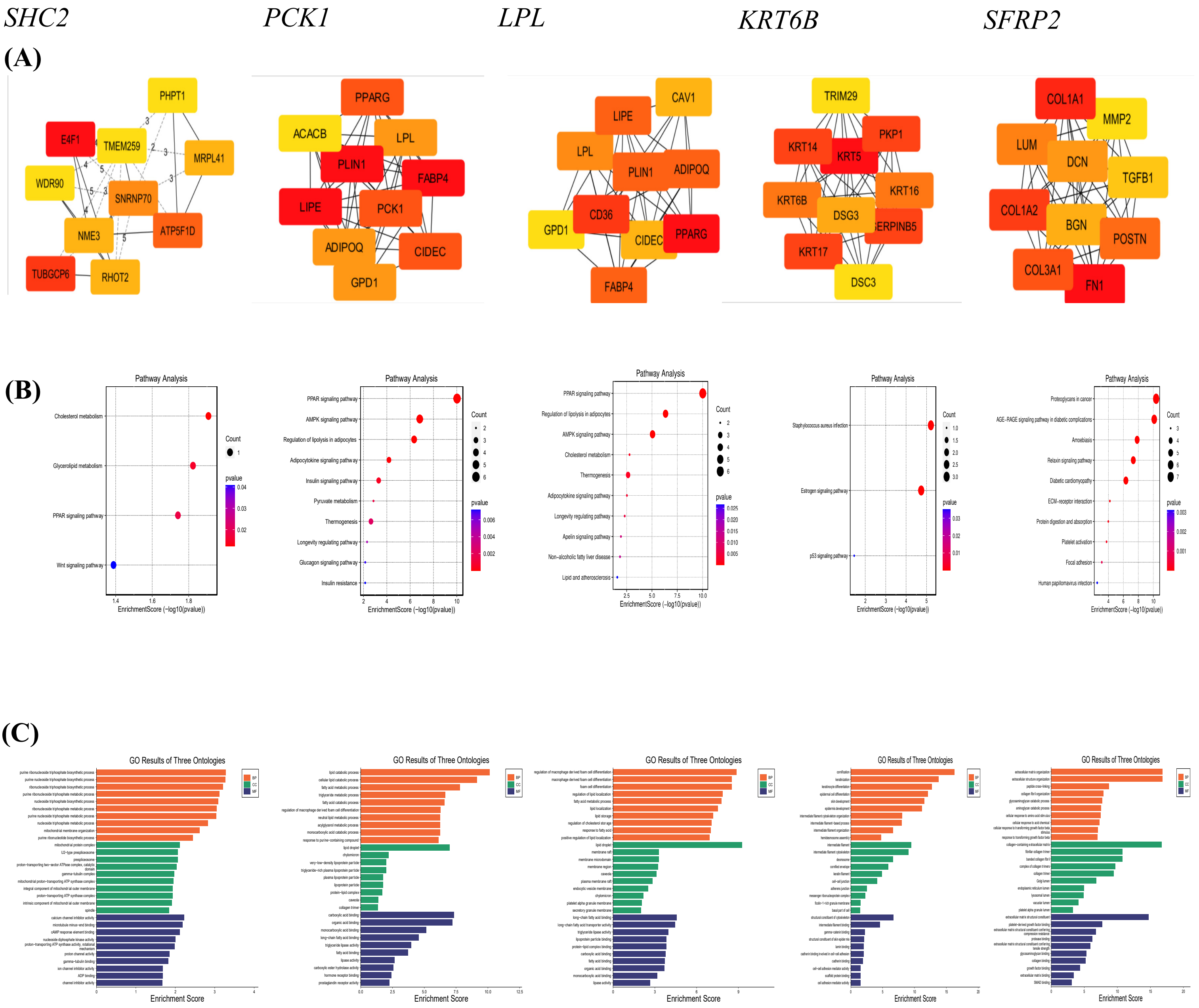

Supplementary Figure S8. Screening results of the function for the key DEGs. A) Top 10 key genes identified by CytoHubba of Degree plug-in. B) Kegg pathway signaling C) Enriched GO terms in the biological process (BP) group, cellular component (CC) and molecular function (MF)

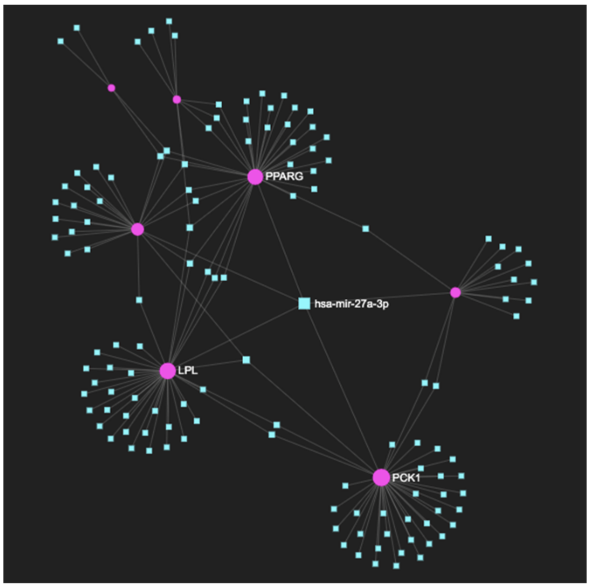

Supplementary Figure S9. Visualization of the interaction network between hub genes and targeted miRNAs using Networkanalyst. The hub genes are shown in purple nodes, whereas targeted miRNAs are shown in blue nodes. The line symbolizes the interaction between the hub genes and related miRNAs

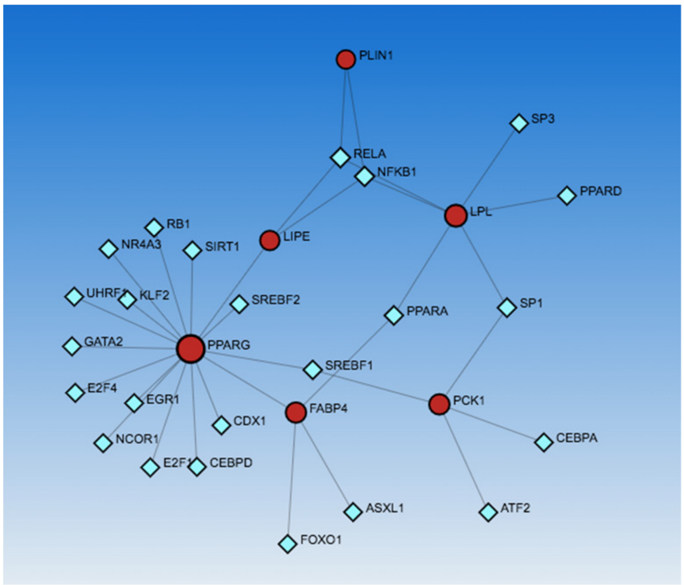

Supplementary Figure S10. Transcriptional regulatory network between the hub genes and the targeted transcriptional factors using Networkanalyst. Blue nodes resemble the transcriptional factor, and the purple nodes represent transcriptional factor regulated hub gene.

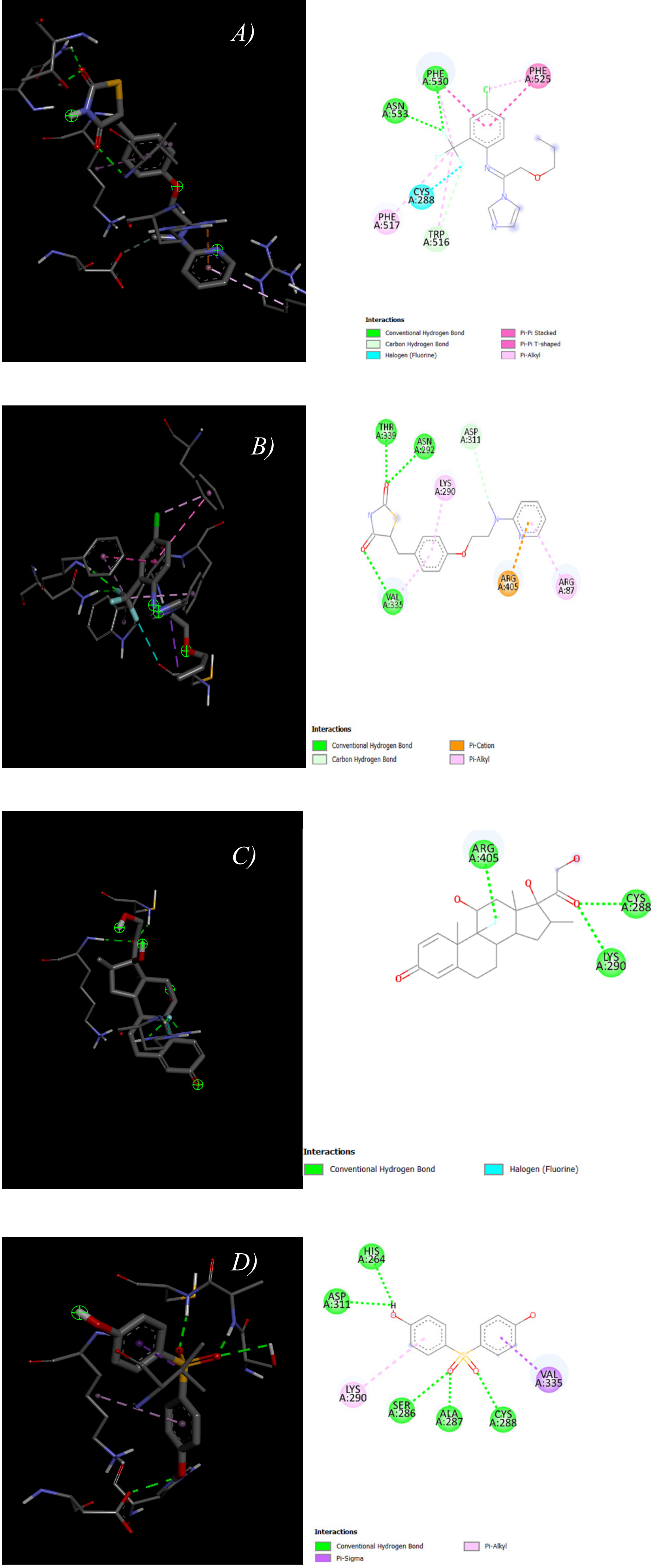

Supplementary Figure S11. The 3D and 2D interaction of 1NHX with (A) Triflumizole (B) Rosiglitazone(C) Dexamethasone (D) bis(*4-hydroxyphenyl*)sulfone

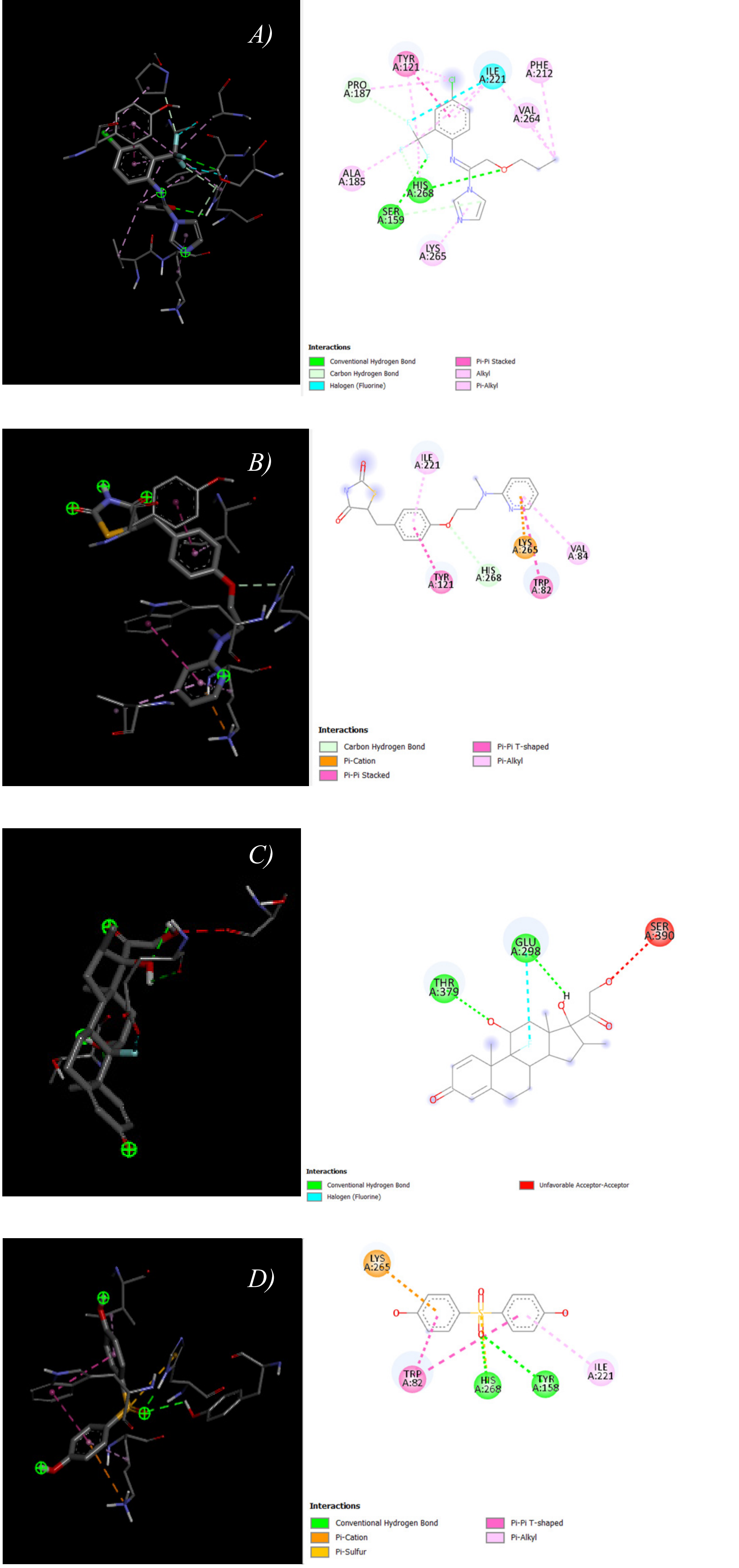

Supplementary Figure S12. The 3D and 2D interaction of 6E7K with (A) Triflumizole (B) Rosiglitazone (C) Dexamethasone (D) bis(*4-hydroxyphenyl*)sulfone

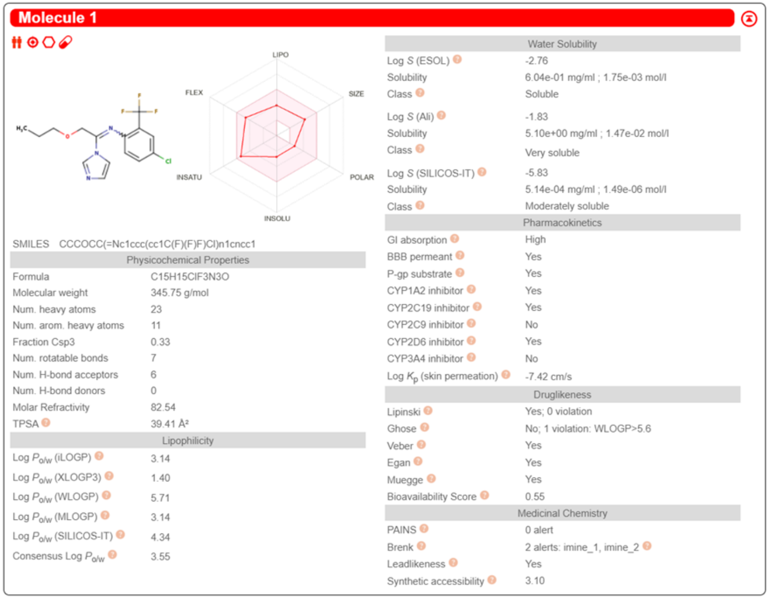

Triflumizole

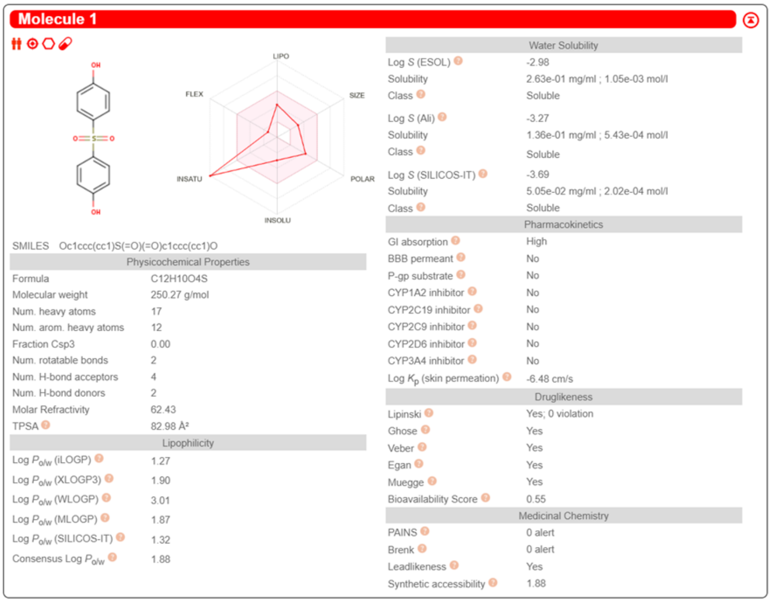

bis(*4-hydroxyphenyl*)sulfone

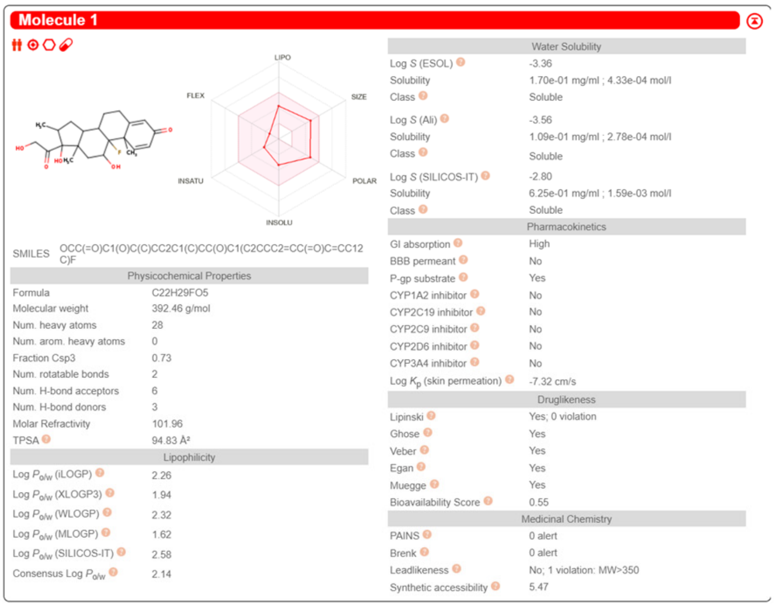

Dexamethasone

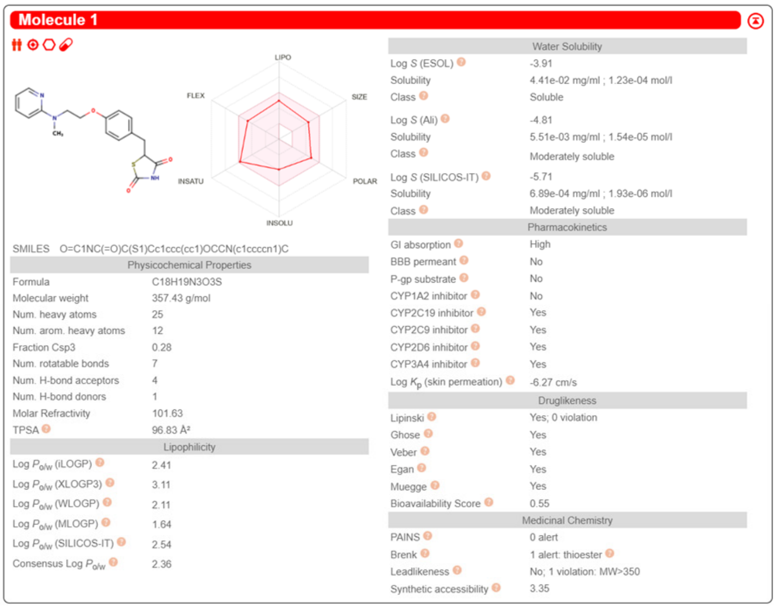

Rosiglitazone

Supplementary Figure S13. SwissADME bioavailability radar of different bioactive drug-likeness molecules

(A)

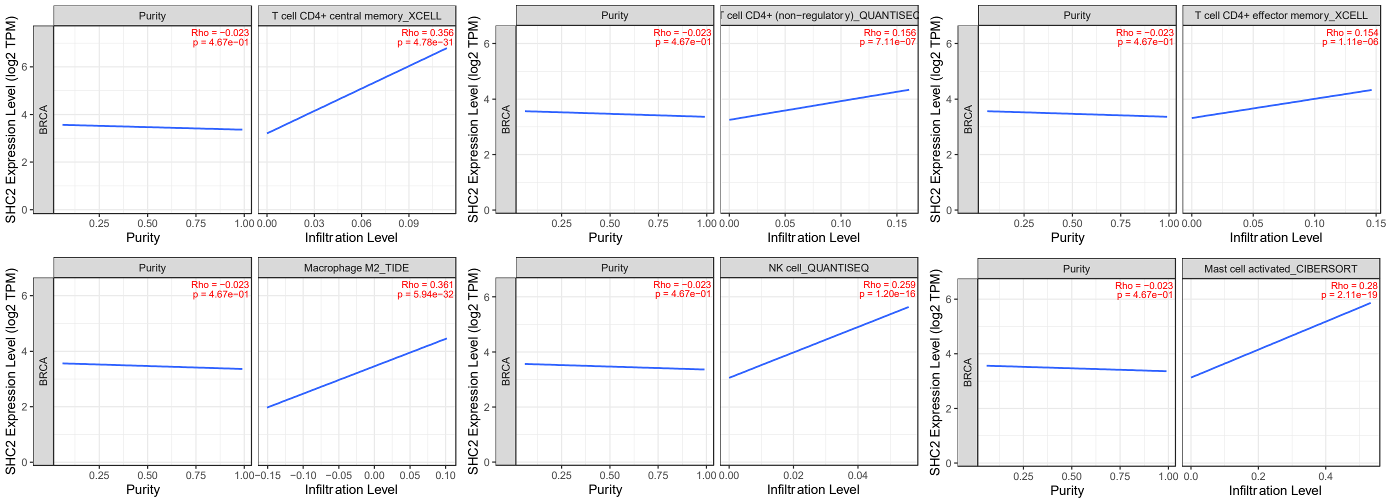

(B)

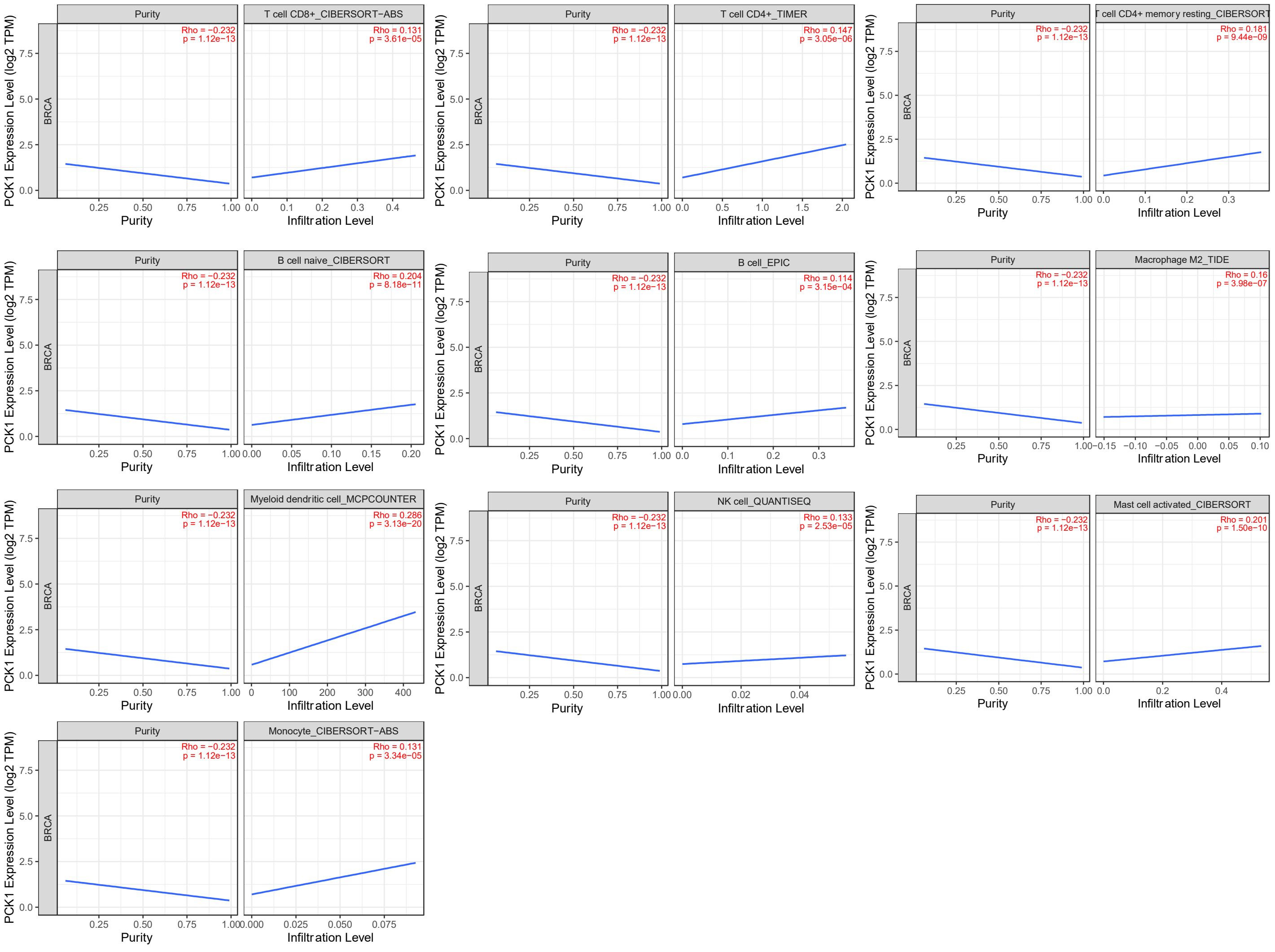

Supplementary Figure S14. Correlation of the expression of two DEGs with immune infiltration level in BRCA using TIMER2.0. (A) Correlation of the expression of SHC2 with immune infiltration level in BRCA. (B) Correlation of the expression of PCK1 with immune infiltration level in BRCA.
