# Supplementary material for: Identification of Genes Crucial for Biological Processes in Breast Cancer Liver Metastasis Relapse
Source: Int J Mol Sci. 2024 May 16;25(10):5439. doi: 10.3390/ijms25105439 (PMC11122209; doi:10.3390/ijms25105439)
Supplement: Supplementary file 1 [file ijms-25-05439-s001.zip › final Supplementary table.pdf]

Supplementary Table S1. List of DEGs responsible for the initiation or the developmental stage of breast cancer identified using the UALCAN database

GENE	Comparison	Statistical significance
PCK1	Normal-vs-Stage1	2.27E-10
	Normal-vs-Stage2	1.69E-10
	Normal-vs-Stage3	2.12E-10
	Normal-vs-Stage4	1.09E-10
	Stage1-vs-Stage4	1.92E-02
	Stage3-vs-Stage4	1.84E-02
LPL	Normal-vs-Stage1	1.63E-12
	Normal-vs-Stage2	3.55E-15
	Normal-vs-Stage3	3.11E-15
	Normal-vs-Stage4	1.44E-15
	Stage1-vs-Stage4	2.58E-03
	Stage2-vs-Stage4	4.06E-03
	Stage3-vs-Stage4	1.68E-02
SFRP2	Normal-vs-Stage1	3.35E-12
	Normal-vs-Stage2	5.88E-15
	Normal-vs-Stage3	9.88E-15
	Stage2-vs-Stage3	1.75E-02
KRT6B	Stage1-vs-Stage2	1.24E-02
	Stage2-vs-Stage3	2.82E-02

Supplementary Table S2. Binding affinities of each chemical towards LPL and PCK1 that were identified using the PyRx software.

GENE	Chemical compounds	Binding affinity
1NHX	Rosiglitazone	-8.4
	Dexamethasone	-8.1
	bis(4-hydroxyphenyl)sulfone	-7.5
	Triflumizole	-7.0
LPL	Rosiglitazone	-7.7
	Triflumizole	-7.5
	bis(4-hydroxyphenyl)sulfone	-7.5
	Dexamethasone	-7.3

Supplementary Table S3. Clinicopathological features of patients from the datasets.

GSE175692	
**Organ**	**Number of Samples**
Breast	16
Liver	26
**Sex**	
Male	0
Female	42
**De novo or relapse**	
De novo	0
Relapse	42
**Distant vs locoregional**	
Distant	30
Local	12
**Pre – post menopausal**	
Pre	18
Post	22
Unknown	**2**
GSE56493	
**Breast**	**19**
Her2	8
Basal	3
LumB	4
LumA	3
Normal	1
**Liver**	**27**
Her2	9
Basal	0
LumB	12
LumA	6
GSE46141	
**Organ**	
Liver	16
Breast	11
